# Rab7a is required to degrade select blood-brain barrier junctional proteins after ischemic stroke

**DOI:** 10.1186/s40478-025-02125-6

**Published:** 2025-09-29

**Authors:** Azzurra Cottarelli, Danny Jamoul, Mary Claire Tuohy, Sanjid Shahriar, Michael Glendinning, Grace Prochilo, Aimee L. Edinger, Ahmet Arac, Dritan Agalliu

**Affiliations:** 1 Department of Neurology, Columbia University Irving Medical Center, New York, NY 10032 USA; 2 Department of Medicine, Columbia University Irving Medical Center, New York, NY 10032 USA; 3 Department of Pathology and Cell Biology, Columbia University Irving Medical Center, New York, NY 10032 USA; 4 Wyss Institute for Biologically Inspired Engineering, Boston, MA 02115 USA; 5 Departments of Developmental and Cell Biology and Pharmaceutical Sciences, University of California, Irvine, CA 92697 USA; 6 Department of Neurology, David Geffen School of Medicine, University of California in Los Angeles, Los Angeles, CA 90095 USA

**Keywords:** Rab7a, Tight junctions, Adherens junctions, Blood-brain barrier, Ischemic stroke, Inflammation, Cytokines, Claudin-5, VE-Cadherin, ZO-1

## Abstract

The integrity of adherens and tight junctions is critical for blood-brain barrier (BBB) function in the healthy brain. Disassembly of cell junctions due to degradation of adherens and tight junction-associated proteins leads to acute BBB dysfunction after ischemic stroke, but the mechanisms of this process are not fully understood. Using genetic studies in mice coupled with histopathological analysis of the brains after ischemic stroke, we demonstrate that endothelial cell deletion of Rab7a, a small GTPase crucial for protein degradation through the endolysosomal system, reduces acute BBB leakage and improves neuronal health in mice after ischemic stroke by reducing the degradation of select adherens and tight junction proteins, and preserving the structural morphology of tight junctions at both confocal and electron microscopy level. Two pro-inflammatory cytokines, TNFα and IL1β, that are known to trigger disruption of paracellular barrier properties in primary brain endothelial cells in vitro and are upregulated after ischemic stroke, contribute to Rab7a activation in primary mouse brain endothelial cells (BECs). In contrast, oxygen-glucose deprivation does not activate Rab7a in mouse BECs. Rab7a is, therefore, critical for degradation of select BEC junctional proteins during the acute increase in BBB permeability after ischemic stroke.

**Supplementary Information:**

The online version contains supplementary material available at 10.1186/s40478-025-02125-6.

## Introduction

The stability of adherens (AJs) and tight junction (TJs) strands formed between brain endothelial cells (BECs) lining the blood vessels is critical for blood-brain barrier (BBB) integrity and together with low rates of transcellular transport, restricts the permeability of blood-derived proteins and immune cells into the healthy central nervous system (CNS; reviewed in [1]). Disassembly of TJ strands associated with BBB damage occurs in several neurological disorders including ischemic stroke, a complex and devastating neurological condition that is the second leading cause of death and the third leading cause of disability worldwide (reviewed in [2]). In addition to the acute damage triggered by BBB breakdown after ischemic stroke (reviewed in [3]), BECs that survive and proliferate in the peri-infarct region of the brain, but do not maintain BBB properties, facilitate further tissue damage and exacerbate the clinical prognosis of stroke leading to post-stroke cognitive dementia [4–6]. Elucidating the mechanisms that promote stabilization of BBB junctions is critical to identify targets that could improve both short- and long-term stroke outcomes. Yet, how AJ and TJ proteins are degraded after ischemic stroke to promote acute BBB damage is not fully understood.

During the reperfusion phase of acute ischemic stroke, there is a biphasic increase in BBB permeability that contributes to vasogenic edema, hemorrhage and increased mortality [7–10]. Although BEC junctional strands are stable in the healthy brain [11, 12], we have shown using green fluorescent protein (GFP)-tagged Claudin-5 that BBB TJs in the cortical penumbra that succumbs later to infarct become highly dynamic and exhibit structural abnormalities between 24 and 58 h after transient middle cerebral artery occlusion (t-MCAO), a rodent model for ischemic stroke [11]. This phase coincides with increased expression of several pro-inflammatory cytokines and recruitment of immune cells into the CNS (reviewed in [13, 14]), that contribute to stroke pathophysiology, including further BBB damage, neuronal death, progression to infarct and worse clinical outcomes [15–17]. What mechanisms drive BBB AJ and TJ disassembly after ischemic stroke? Increased expression and activity of several matrix metalloproteases (MMPs; e.g., MMP2 and MMP9) after ischemic stroke contributes to acute BBB damage by cleaving the extracellular domains of several adherens (VE-Cadherin) and tight (Claudin-5, Occludin) junction proteins in BECs (reviewed in [18, 19]). Ubiquitin-mediated proteasome degradation has also been proposed as a mechanisms for degradation of transmembrane proteins (VE-Cadherin, Claudin-5 and Occludin) and intracellular adaptors (ZO-1 and β-catenin) that anchor transmembrane junctional proteins to the cytoskeleton in various diseases including ischemic stroke (reviewed in [20, 21]). Finally, caveolae have also been proposed to promote internalization and degradation of TJ transmembrane proteins such as Claudin-5 and Occludin in brain and peripheral ECs in vitro [22]. However, *Caveolin-1 (Cav-1)*^−/−^ mice lacking caveolae [23, 24] show a similar degree of paracellular BBB damage, although a reduced transcellular BBB damage, and similar infarct size to wild-type mice after t-MCAO [11, 25], suggesting a caveolar-independent mechanism for BBB AJ and TJ degradation.

Enhanced endocytotic degradation of BBB junctional proteins in response to the inflammatory milieu is another potential mechanism for persistent AJ and TJ strand disassembly post-ischemic stroke. EC junctional strand protrusions express the early endosomal antigen EEA-1 [12] and they are inhibited by treatment with inhibitors of endocytosis [26]. During endocytosis, early endosomes and their cargoes can either be sorted back to the plasma membrane as recycling endosomes, or directed to degradation by maturing into late endosomes that ultimately fuse with lysosomes [27]. The small GTPase Rab7a (also known as Rab7) is critical for sorting cargoes into the late endosome, biogenesis of lysosomes and phagocytosis, as well as regulation of substrate degradation, antigen presentation, cell signaling/survival and microbial infection [28, 29]. Although the endolysosomal functions of Rab7 have been studied in healthy mammalian cells, how its activity is regulated in neurological diseases is not well understood. Growth factor deprivation in cancer cells increases the fraction of Rab7a associated with membranes, and Rab7a-GTP (active form) triggers cell death due to degradation of nutrient transporters essential for survival [30–32]. Conversely, Rab7a inhibition after growth factor withdrawal promotes cancer cell survival due to recycling of transporters to the cell surface [33, 34]. Regulatory proteins that control Rab7a activity have been difficult to identify in many cells. TBC1D15 has been identified as a putative Rab7a inactivator (GAP), since it accelerates GTP hydrolysis by Rab7a in vitro and its overexpression disrupts lysosomal morphology and blocks growth factor withdrawal-induced death in cancer cells [33]. Ccz1, a guanidine exchange factor (GEF) that acts together with vacuolar fusion protein Mon1, has been shown to promote Rab7a activation and Rab7a-mediated membrane fusion in yeast [35] and eukaryotic cells [36–39]. Rab5a and Rab7a regulate VEGFR2 receptor trafficking and the response of ECs to VEGF-A [40] via the Rab effector protein RABEP2 [41]. The liver kinase B1 (LBK1) has also been identified as a Rab7a effector in ECs that promotes Neuropilin-1 receptor trafficking and degradation to inhibit angiogenesis [42]. Moreover, Rab4 activation and Rab9 inhibition are also critical for the vascular permeability in lung ECs, through regulation of VE-Cadherin localization to cell junctions [43]. Yet, how endothelial Rab7a contributes to acute BBB dysfunction after ischemic stroke is unknown.

Here, we show that endothelial-specific deletion of Rab7a improves neuronal survival and reduces acute BBB disruption in mice after ischemic stroke by preventing degradation of some junctional proteins and preserving TJ morphology at both confocal and electron microscopy levels. Two pro-inflammatory cytokines, TNFα and IL1β, that are known to trigger disruption of paracellular barrier properties in primary brain endothelial cells in vitro [12] and are upregulated after ischemic stroke [14], contribute to Rab7a activation in primary mouse brain endothelial cells (BECs). In contrast, oxygen-glucose deprivation does not activate Rab7a in BECs. These findings identify Rab7a as a critical regulator of select junctional protein degradation during the acute BBB damage following ischemic stroke.

## Materials and methods

### Mice

All experimental procedures in mice were approved by the IACUC committees at the University of California, Irvine and Columbia University Irving Medical Center. The following mouse strains were used: *Tg(eGFP-Claudin5)* [11]; *Rab7a*^*fl/fl*^ [34]; *Cdh5(PAC)-Cre*^*ERT2*^*(VEC-PAC)* [44]. To induce Cre^ERT2^-mediated recombination, 4-OH-tamoxifen (Sigma-Aldrich) was dissolved in 10% ethanol/corn oil mixture to a final concentration of 2 mg/ml, and administered by intraperitoneal injections at a dose of 35 µg/g body weight for 5 consecutive days starting at postnatal day P1 - P5 and at the same dose (35 µg/g) for 5 consecutive days in adult mice ending one week before the t-MCAO procedure. This regimen was used to ensure Rab7a gene ablation in all endothelial cells since it has been reported that mosaic deletion of genes in CNS endothelial cells gives advantage to wild-type compared to mutant endothelial cells over time [45]. Despite this unusual regimen of 4-OH tamoxifen administration, we have not observed any toxicity effects in mice, and there is no effect on BBB integrity in healthy *Rab7a*^*iECKO*^ compared to *Rab7a*^*fl/fl*^ (controls).

### Reagents

**Table:** 

Reagent or resource	Source	Identifier
**Primary antibodies**		
Rabbit anti-Rab7a	Abcam	Cat #ab137029 RRID: AB_2629474
Mouse anti-ZO-1	Thermo Fisher Scientific	Cat #33-9100, RRID: AB_2533147
Rabbit anti-ZO-1	Thermo Fisher Scientific	Cat# 61-7300, RRID: AB_2533938
Rabbit anti-GLUT1	Millipore	Cat# 07-1401, RRID: AB_1587074
Rabbit anti-Claudin-5	Zymed	Cat #34-1600, RRID: AB_2533157
Rabbit anti-VE-Cadherin	Abcam	Cat# ab33168, RRID: AB_870662
Rat anti-CD68	Abcam	Cat# ab53444, RRID: AB_869007
Rabbit anti-Iba1	FUJIFILM Wako Shibayagi	Cat# 019-19741, RRID: AB_839504
Mouse anti-NeuN	Sigma-Aldrich	Cat #MAB377, RRID: AB_2298772
Mouse anti-Beta-actin	Novus	Cat# NB600-501, RRID: AB_10077656
Rabbit anti-Caveolin 1	Abcam	Cat# ab18199, RRID: AB_444314
Mouse anti-GST	Santa Cruz Biotechnology	Cat# sc-57,590, RRID: AB_783590
Rabbit anti-Occludin	ThermoFisher Scientific	Cat# 71-1500, RRID: AB_2533977
Goat anti-GFAP	Abcam	Cat# ab53554, RRID: GR3178754-3
Rat anti-VE-Cadherin	BD Pharmigen	Cat# 555,289 RRID: 3,086,096
Mouse anti-ZO-1	Invitrogen	Cat# 33-9100 RRID: YK381714
Rabbit anti-Claudin-5	Invitrogen	Cat# 34-1600 RRID: VC299747
Rabbit anti-Glut1	EMD Millipore Corp.	Cat# 07-1401 RRID: AB_4124999
Rabbit anti-Glut1	Abcam	Cat# ab652 RRID: GR3213004-1
Rabbit anti cleaved Caspase-3	Cell Signaling Technology	Cat # 9664 S RRID: AB_2070042
**Chemicals**,** peptides**,** and recombinant proteins**		
Biocytin-TMR (5-(and-6)-tetramethylrhodamine biocytin)	ThermoFisher Scientific	Cat #T12921
Albumin from Bovine Serum (BSA), AlexaFluor™ 594 conjugate	ThermoFisher Scientific	Cat #A13101
Griffonia (Bandeiraea) Simplicifolia Lectin I (GSL I, BSL I), Rhodamine	Vector Labs	Cat #RL-1102-2
Recombinant Mouse TNFα	R&D systems	Cat # 410-MT
Recombinant Mouse IL-1β	R&D systems	Cat #401-ML
**Experimental Models**		
C57BL/6 mouse primary brain microvascular endothelial cells	Cell Biologics	Cat #C57-6023
*Rab7a* ^*flox/flox*^	Dr. Aimee Edinger	RRID: IMSR_JAX:021589
*Cdh5(PAC)-Cre* ^*ERT2*^ *(VEC-PAC)*		RRID: IMSR_GPT: T052686
*Tg(eGFP-Claudin5)*		Published in [11]
**Plasmids**		
pGEX-4T-3-mR7BD	Addgene	Cat #79,149, RRID: Addgene_79149
**Oligonucleotides**		
*Rab7a flox* forward: 5’-CTCACTCACTCCTAAATGG-3’	IDT	N/A
*Rab7a flox* reverse: 5’-TTAGGCTGTATGTATGTGC-3’	IDT	N/A
*Rab7a null* forward: 5’-GGGCTGCAGGAATTCGGATAAC-3’	IDT	N/A
*Rab7a null* reverse: 5’-CATGGTAACAAGTCTGTCGTCC-3’	IDT	N/A
*Cre* forward: 5’-GCTAAGTGCCTTCTCTACACCTGC-3’	IDT	N/A
*Cre* reverse: 5’- GGAAAATGCTTCTGTCCGTTTG − 3’	IDT	N/A
*GFP* forward: 5’-CCCTGAAGTTCATCTGCACCAC-3’	IDT	N/A
*GFP* reverse: 5’-TTCTCGTTGGGGTCTTTGCTC-3’	IDT	N/A
**Software and algorithms**		
Zen microscopy software	Zeiss	RRID: SCR_013672
Prism 9	GraphPad	RRID: SCR_002798
Empiria Studio	LI-COR	RRID: SCR_022512
ImageJ/Fiji	NIH	RRID: SCR_002285

## Methods

### Mouse ischemic stroke model

Ischemic stroke was induced in 12–14 weeks old male mice by t-MCAO for 45 min as described [46]. Briefly, mice were anesthetized with 1.5-2% isoflurane, left external and common carotid artery were ligated and a 7 − 0 silicon rubber-coated monofilament (Doccol) was inserted through the common carotid artery into the internal carotid artery. Reperfusion was obtained by removing the monofilament 45 min after its insertion. The body temperature of the mice was monitored and maintained at 37 °C throughout the procedure. The neurological deficits in mice after t-MCAO were evaluated and scored as described in Jiang S.X. et al., 2005 [47].

### Biocytin-TMR, 70 kda dextran-TMR and serum IgG permeability analysis

Mice received a tail vein injection of 100 µl of biocytin-TMR (1% in PBS; ThermoFisher Scientific) or 70 kDa dextran-TMR (1% in PBS, Thermofisher Scientific). The dye was allowed to circulate for 30–45 min. For biocytin-TMR leakage analysis, mice were anesthetized with isoflurane and perfused first with PBS, then with 4% paraformaldehyde (PFA) in PBS. AlexaFluor 488-conjugated goat anti-mouse IgG (Invitrogen, 1:500) was used to visualize serum IgG leakage in brain sections. The analysis of biocytin-TMR or serum IgG leakage was performed as described before [11]. Briefly, brain sections located at seven different distances from the bregma landmark were imaged with an upright Zeiss Axioimager fluorescence microscope. Biocytin or IgG leakage was quantified with Fiji software. Brain slices were uniformly thresholded in order to quantify the total area of IgG or biocytin-TMR leakage. Areas that exceeded the threshold levels were defined as leakage area. Average intensity values for biocytin-TMR were gathered by selecting identical regions in ipsilateral or contralateral cortices or livers across animals, then the ratio between the ipsilateral and contralateral cortex was determined. For 70 kDa dextran-TMR analysis, the brains were collected from anesthetized, but not perfused, mice, the ipsilateral and contralateral cortexes were dissected out from the rest of the brain, the tissue was homogenized in PBS with a douce homogenizer, the homogenate was centrifugated at 13,000 rpm for 30 min, and the tracer was extracted in PBS as described [48]. The measurement of the fluorescence from the tissue and quantification were done using a 96-well black plate in an AccuScanFC plate reader (ThermoFisher) with the excitation/emission filters values of 550/570 to detect tetramethylrhodamine (TMR) as described [48].

### Immunofluorescence staining

The brains and livers were dissected from PFA-perfused mice, fixed in 4% PFA at 4 °C for 6 h, washed three times with PBS for 30 min per wash, cryoprotected in 30% sucrose/PBS overnight and embedded in Tissue-Tek / OCT. Brains were sectioned in 12 μm-thick coronal slices spanning all distances from the bregma landmark using a Leica cryostat. For immunofluorescence staining of some proteins (e.g. Rab7a) brains were dissected after perfusion of mice with PBS and fresh-frozen in Tissue-Tek/ OCT. Brain sections were fixed with cold 95% ethanol for 30 min and acetone for 1 min followed by three 5-minute washes with PBS. For immunofluorescence staining of tight junction proteins (Claudin-5, ZO-1) in PFA-fixed brain sections, antigen retrieval was performed with 1X citrate buffer (pH = 6, Millipore, Cat # C9999) warmed to 100 °C in a water bath for 25 min then cooled to room temperature. The following primary antibodies were used for immunofluorescence staining of brain sections: rabbit anti-Rab7a (1:100, Abcam), goat anti-GFAP (1:250, Abcam), rat anti-VE-cadherin (1:25, BD Pharmigen), mouse anti-ZO-1 (1:500, Invitrogen), rabbit anti-ZO-1 (1:500, Invitrogen), rabbit anti-GLUT1 (1:200, Thermo Scientific), rabbit anti-Claudin5 (1:500, Invitrogen), rabbit anti-VE-cadherin (1:250, Abcam), rat anti-CD68 (1:500, Abcam), rabbit anti-Iba1 (1:1000, Wako), mouse anti-NeuN (1:500, Millipore), rabbit anti cleaved Caspase 3 (1:500, Cell Signaling). BSL-rhodamine (1:250, Vector Laboratories) was used to label the vasculature. The following goat or donkey secondary antibodies generated against a specific primary antigen (rabbit, mouse, rat or goat) were used for immunofluorescence staining: goat anti-rat Alexa Fluor 594 (A11007), goat anti-rabbit Alexa Fluor 594 (A11012), goat anti-rat Alexa Fluor 488 (A11006), goat anti-rabbit Alexa Fluor 488 (A11034), goat anti-mouse Alexa Fluor 488 (A11001), donkey anti-rat Alexa Fluor 594 (A21209), goat anti-mouse Alexa Fluor 594 (A11032), donkey anti-rabbit Alexa Fluor 594 (A32754), donkey anti-rat Alexa Fluor 488 (A21208), donkey anti-rabbit Alexa Fluor 488 (A21206), donkey anti-rat Alexa Fluor 647 (A48272), donkey anti-rabbit Alexa Fluor 647 (A32795), donkey anti-goat Alexa Fluor 594 (A32758) and donkey anti-goat Alexa Fluor 488 (A11055) (Thermo Fisher Scientific). The secondary antibodies were diluted either at 1:1000 (Alexa Fluor 488 or 594) or 1:500 (Alexa Fluor 647) and incubated with the tissue sections or cells for 2 h at room temperature.

### Western blotting

#### Cortical lysates

Ipsilateral and contralateral cortexes of mice were dissected from the rest of the brain 48 h after t-MCAO. The tissue was placed in RIPA buffer with protease and phosphatase inhibitors and homogenized with a Dounce homogenizer. The homogenized tissue was centrifuged for 30 min (13000 rpm). Total protein levels were assessed with the BSA protein assay (Pierce BCA Protein Assay). The samples containing 40 µg of total protein were denatured at 95 C for 5 min and ran on a 4–15% denaturing gel (Biorad) at 150 V for 30–45 min. The proteins were then transferred into an Immobilon PVDF membrane at 100 V for 95 min. The following primary antibodies were used: mouse anti-Claudin5 (1:500, Invitrogen), rabbit anti-Claudin-5 (1:500, Zymed) rabbit anti-Caveolin-1 (1:2000, Abcam), rabbit anti-Occludin (1:500, Thermofisher), rabbit anti-VE-cadherin (1:500, Abcam), mouse anti-ZO-1 (1:500, Invitrogen), rabbit anti-Rab7a (1:1000, Abcam), mouse anti-GST (1:1000, Santa Cruz), mouse anti-β-actin (1:10000, Novus Biologicals). IR-Dyes 680 and 800 (1:10000, LI-COR) were used as secondary antibodies. Protein levels for endothelial markers were assessed using the Odyssey Sa infrared imaging system (LI-COR). β-actin was used as housekeeping gene. Protein expression levels were quantified using LI-COR Biosciences Image Studio software (version 5.2, 2015) and Empiria Studio software (version 3.2.0.186, 2024), normalized on their respective β-actin expression values and presented as a percentage of protein levels in control conditions.

### mBEC culture and Rab7a-GTP pull-down assay

Primary mBECs were purchased from Cell Biologics and grown in endothelial cell medium supplemented with growth factors and 5% FBS (Cell Biologics). The plasmid pGEX-4T-3-mR7BD, which expresses a recombinant protein consisting of the Rab7a binding domain of the murine RILP protein fused to the C terminus of GST (Addgene, Cat#79149), was transformed into Escherichia coli strain BL21. The transformed bacteria were induced to express the GST-RILP protein, which was then purified from the bacterial cell lysates using glutathione-Sepharose 4B beads (GE Healthcare) as described [31]. The quality of the GST-RILP fusion protein was verified by performing the Rab7a pulldown assay as described before [31, 49] using 600 µg of total protein lysates from HEK293 cells transfected with plasmids for the constitutively active form (Rab7Q76L), or inactive form (Rab7 T22N) form of human Rab7a protein as described [49]. Once confirmed, the GST-RILP protein was used for the Rab7a pulldown assay in primary mBECs. Control, cytokine- or OGD-treated mBECs were lysed in pull-down buffer (20 mM HEPES, 100 mM NaCl, 5 mM MgCl_2_, 1% TX-100, and protease inhibitors) and sonicated. Protein levels in the mBEC lysates were quantified via the BCA assay. Each pull-down was performed by incubating 30 µl of the GST-RILP bound bead slurry pre-equilibrated in pull-down buffer with 600 µg of total protein lysates from either condition, and rocking the bead-lysate mixture in a nutator overnight at 4 °C described before [31, 49]. Afterwards, the beads were washed three times with cold pull-down buffer, and bound proteins were eluted by adding sample loading buffer with SDS and incubating at 95 °C for 10 min. The Western blotting for the Rab7a protein (active form) and GST were performed from the eluates and for total Rab7a and β-Actin were performed from the initial protein lysates as described above.

### Microscope image acquisition and quantification

Images of the whole brain sections were acquired with a Zeiss Axioimager fluorescence microscope, higher resolution images were acquired using a Zeiss LSM700 confocal microscope. For comparison purposes, all images of the same staining were acquired under the same settings. All images were processes using Fiji, and all quantifications were carried out blinded. The areas of Biocytin-TMR or IgG leakage were quantified by uniformly thresholding the brain sections and measuring the area exceeding the threshold in each section. The volume of the leakage was calculated as the product of cross-sectional areas and distance between sections. Intensity of the leakage was determined by sampling identical regions in contralateral and ipsilateral cortex in each animal, normalizing their fluorescence intensity on the average fluorescence intensity of the liver from the same animal and calculating the ratio between the ipsilateral and the contralateral cortex.

To quantify junctional abnormalities, vessel segments with intact TJ strands, TJ strands containing gaps or vessel segments without any TJ strands (absent) were identified as described before [11] from 5 to 6 independent ROIs within a specific region and the percentage of vessel segments with either intact TJ strands, TJ strands with gaps or absent junctional strands over the total number of vessel segments was calculated per image in ImageJ. We defined junctional status along a Glut-1-positive endothelial cell mask as follows: a) a “gap” required a continuous Glut1-positive segment in which ZO-1 intensity fell below a pre-specified threshold while Glut1 staining remained intact. Quantification of the TJ strand gaps was restricted to the Glut1 + membrane mask and puncta off the membrane did not influence the scoring. The vessels coverage of junctional strands per each ROI was calculated by thresholding the junctional marker within the vascular marker after masking in ImageJ. Since this analysis samples the signal along the Glut-1-defined endothelial cell mask, a single-plane confocal imaging at the junctional plane is sufficient for this membrane-referenced analysis. To quantify neuronal viability, we calculated the ratio of NeuN positive cells with intact NeuN staining over the total number of cells visualized with DAPI for each field of view in the ipsilateral or contralateral cortex. The “ipsilateral” ROIs were acquired in the ipsilateral cortical regions that showed both serum IgG leakage and biocytin-TMR leakage in both genotypes. This region becomes infarcted with the infarct growth in wild-type mice, but not completely infarcted in *Rab7a*^*iECKO*^ mice.

### Transmission electron microscopy

Mice were perfused with PBS for 5 min. The ipsilateral or contralateral cortices were dissected from the perfused brains and placed in fixative solution (4% PFA and 2% glutaraldehyde in 0.1 M sodium cacodylate) overnight at 4 °C, followed by washing with buffer (0.1 M sodium cacodylate) and water. Fixed samples were treated with 1% reduced Osmium Tetroxide, dehydrated in an ethanol series, followed by treatment with acetonitrile. Samples were then embedded in LX112 resin (Ladd Research Industries) and sectioned. Sections were contrasted with uranyl acetate and lead citrate for imaging in JEOL JEM-1400 TEM equipped with a Veleta (EMSIS, GmbH) CCD camera in the Microscopy and Image Analysis Core Facility, Weill Cornell Medicine (New York, NY, USA). We acquired 16–22 images in each ipsilateral cortex (*n* = 3 mice / genotype) and analyzed 46–55 tight junctions per mouse.

### Statistical analyses

All statistical analyses were performed using GraphPad Prism version 8.1 or higher. Unless differently specified in the figure legend, data are represented as mean ± s.e.m. The Shapiro-Wilk test was used to verify the normality of all datasets. For dataset showing normal distribution, pairwise comparisons were performed using a two-tailed Student’s t-test and multiple comparisons were performed using ordinary one-way ANOVA with Tukey’s multiple comparison test. *P* values lower than 0.05 were considered statistically significant (***: *p* < 0.001; **: *p* < 0.01; *: *p* < 0.05).

## Results

### Rab7a deletion in endothelial cells reduces acute paracellular BBB permeability 48 h after t-MCAO

**Fig. 1:**
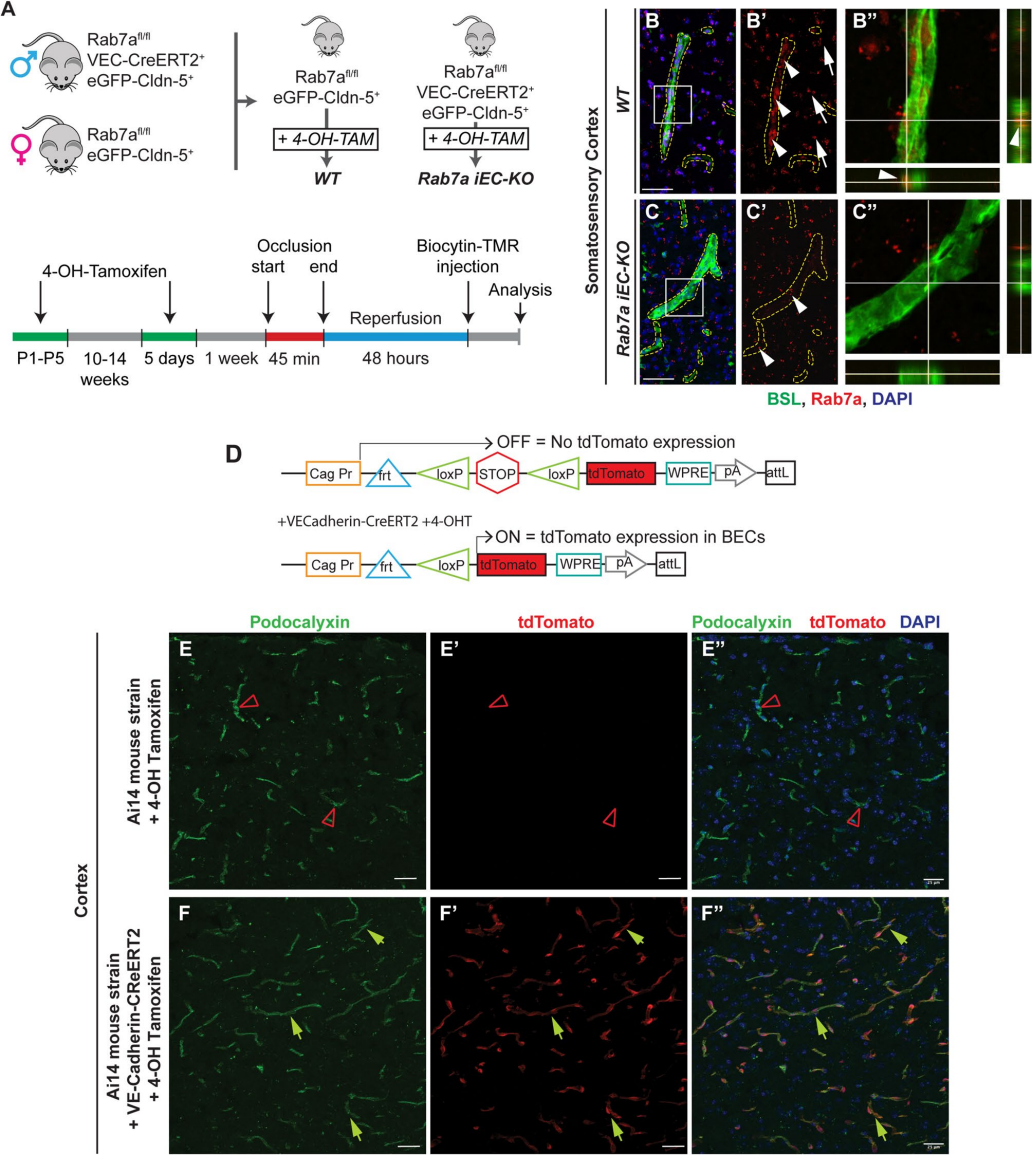
Generation of endothelial-specific Rab7a deficient mice. (**A**) Diagrams of the breeding strategy to generate the inducible endothelial Rab7a knockout (*Rab7a*^*iECKO*^) mice and the experimental setup to analyze blood-brain barrier (BBB) permeability after t-MCAO. (**B-C”**) Immunofluorescence images for Griffonia (Bandeiraea) Simplicifolia Lectin I (BSL, green), Rab7a protein (red) and DAPI (blue) in the cortex of healthy *Rab7a*^*fl/fl*^ (WT, **B-B”**) and *Rab7a*^*iECKO*^ (**C-C”**) mice. Cortical vessels are delineated by yellow dotted lines (**B’**, **C’**). Boxed areas are magnified in the orthogonal view images on the right (**B”**, **C”**). Arrowheads indicate endothelial Rab7a, arrows indicate non-endothelial Rab7a. There is no Rab7a protein in brain endothelial cells of mutant mice. Scale bar = 30 μm. **(D)** Schematic diagram of Ai14 ROSA reporter mice containing a *loxP-*flanked STOP site before tdTomato to visualize the Cre-mediated recombination efficiency following breeding with the *VE-Cadherin-Cre*^*ERT2*^ mice and 4-OH tamoxifen administration. **(E-F’)** Immunofluorescence images for Podocalyxin (vessel marker, green), tdTomato (red), and DAPI (blue) in the cortex of Ai14 mice and Ai14 + *VE-Cadherin-Cre*^*ERT2*^ mice show that tdTomato is expressed in all blood vessels following Cre-mediated recombination induced by 4-OH tamoxifen. Normal green arrowhead corresponds to vessels expressing tdTomato, red open arrowhead indicates vessels not expressing tdTomato. Scale bar = 23 μm

Rab7a inhibition is critical to prevent degradation of nutrient transporters and support survival in lymphocytes and cancer cells [31, 33, 34]. To determine if Rab7a may similarly mediate degradation of BBB TJ proteins in BECs after ischemic stroke, we generated a Rab7a-inducible EC knockout mouse strain [*Rab7a*^*iECKO*^*(Rab7a*^*fl/fl*^; *VEC-Cre*^*ERT2+/−*^; *eGFP::Claudin5*^*+/−*^] to ablate Rab7a in ECs upon administration of 4-OH-tamoxifen (Fig. 1A). These mice were crossed to eGFP::Claudin5 transgenic mice [11] to visualize BBB TJ strand morphology after ischemic stroke. Rab7a protein expression was completely abolished in Lectin (BSL)^+^ cortical blood vessels, but not other CNS cell types, of *Rab7a*^*iECKO*^ compared to *Rab7a*^*fl/fl*^ (*Rab7a*^*fl/fl*^; *eGFP-Claudin5*^*+/−*^; referred as WT) cortices by immunofluorescence (Figs. 1B-C**”**), confirming the efficiency and specificity of Rab7a elimination in BECs. We also analyzed the efficiency of 4-OH tamoxifen-induced Cre deletion in BECs by crossing the *VEC-Cre*^*ERT2+/−*^ strain with an Ai14 reporter mouse strain and confirmed that all CNS blood vessels expressed tdTomato after 4-OH tamoxifen administration (Fig. 1D-F**”**, yellow arrows).

**Fig. 2:**
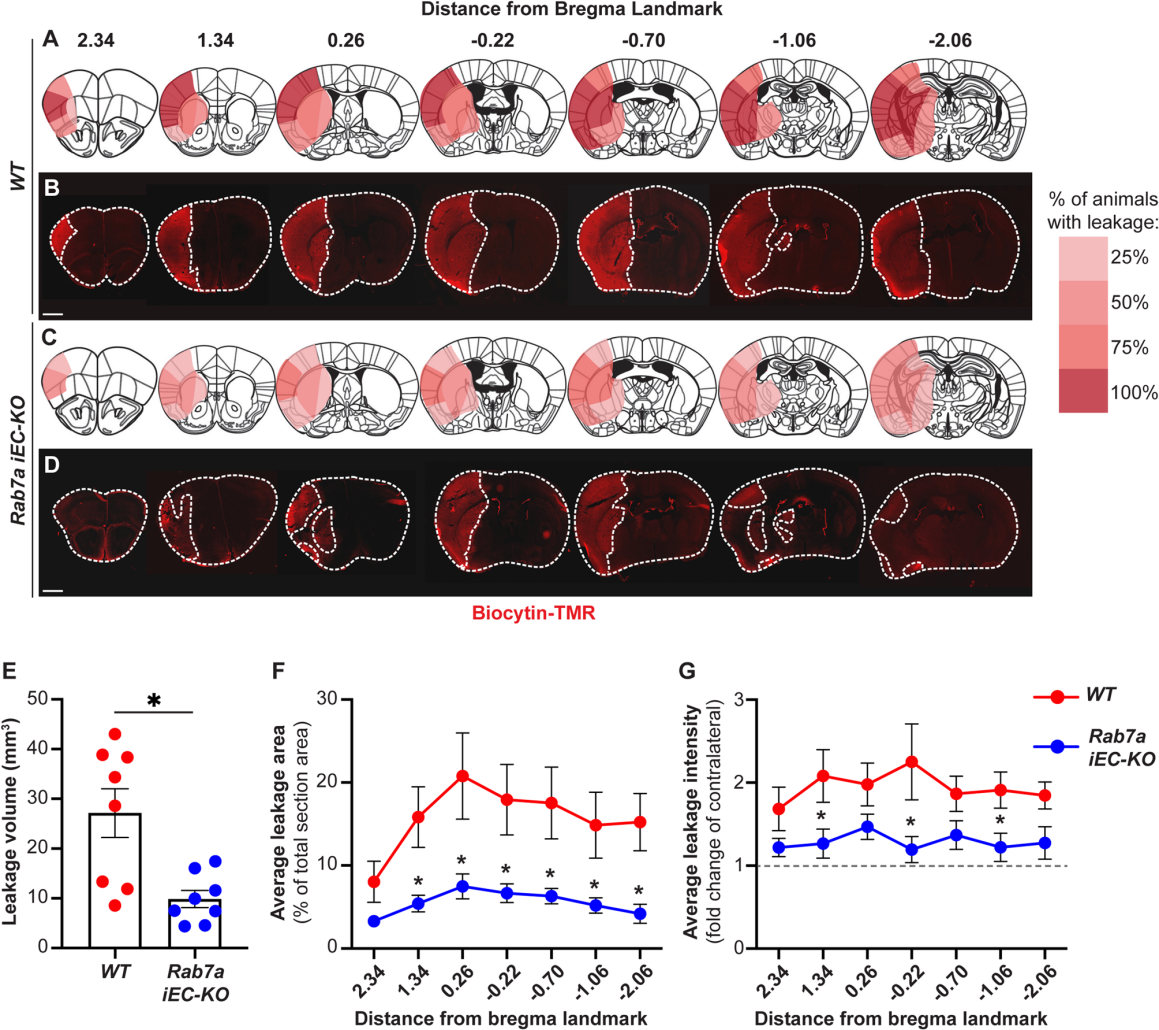
*Rab7a*^*iECKO*^ mice show a partial rescue in the paracellular BBB permeability 48 h after t-MCAO. (**A-D**) Fluorescent micrographs and heatmaps showing biocytin-TMR tracer extravasation in seven brain sections located at different distances from the bregma landmark in WT (*Rab7a*^*fl/fl*^) (**A**,** B**) and *Rab7a*^*iECKO*^ (**C**,** D**) mice 48 h after t-MCAO (biocytin-TMR was injected 30–45 min before analysis). Dotted lines outline the border of the brain section and the leakage area. The heatmaps show the fraction of animals displaying BBB leakage in each region represented as a scale (0-100%) of red hues. (**E**) Quantification of biocytin-TMR leakage volume in the brain of WT and *Rab7a*^*iECKO*^ mice 48 h after t-MCAO (*n* = 8 animals/group). Data are means ± s.e.m. *: *p* < 0.05; Student’s t-student. (**F**,** G**) Quantification of biocytin-TMR leakage area (**G**) and intensity (**I**) in seven brain sections located at different distances from the bregma landmark of WT and *Rab7a*^*iECKO*^ mice 48 h after t-MCAO. The dotted line represents the average fluorescence intensity in the contralateral cortex in **G**. Each dot represents the average of *n* = 8 animals / group. Data are means ± s.e.m. *: *p* < 0.05; one-way ANOVA with post-hoc Tukey’s correction

We induced ischemic stroke in *Rab7a*^*fl/fl*^ and *Rab7a*^*iECKO*^ mice by performing t-MCAO for 45 min [11] and analyzed the effect of Rab7a EC-specific elimination on acute changes in paracellular BBB permeability at 48 h after ischemic stroke following injection of a small dye biocytin-tetramethyl rhodamine (biocytin-TMR; 890 Da) that preferentially extravasates from blood vessels through the paracellular route [11, 12]. *Rab7a*^*fl/fl*^ mice [referred to as wild-type (WT) mice] showed an intense biocytin-TMR fluorescence signal in the ipsilateral cortex and striatum in all brain sections located at seven different distances from the bregma landmark after stroke (Fig. 2A, B). In contrast, the extravascular tracer leakage was less extensive in the ipsilateral cortex and striatum of matched brain sections from *Rab7a*^*iECKO*^ mice (Fig. 2C, D). The average leakage area, reflective of BBB disruption, was significantly reduced in *Rab7a*^*iECKO*^ compared to WT brains across all sections sampled at seven different distances from the bregma landmark, corresponding to ~ 2.5 fold decrease in the overall volume of tracer leakage (Fig. 2E, F). In addition, the brain area affected by BBB impairment was reduced by ~ 2.0-2.5 fold in some brain sections of *Rab7a*^*iECKO*^ mice, and the amount of tracer leakage, measured as mean fluorescence intensity of the extravasated dye in the parenchyma, was also significantly reduced in *Rab7a*^*iECKO*^ mice (Fig. 2F, G). Thus, Rab7a EC-specific deletion rescues partially the acute increase in paracellular BBB permeability at 48 h after t-MCAO.

We have previously shown that the increased BBB permeability during the reperfusion phase after t-MCAO is mediated by two independent cell biological mechanisms: (1) an initial upregulation of endothelial transcytosis that accounts for the early peak in enhanced BBB permeability, and (2) delayed disassembly of BBB adherens and tight junction protein complexes in the second phase that exacerbates BBB dysfunction [11]. To determine if Rab7a deletion in ECs impacts transcellular BBB permeability after ischemic stroke, we analyzed the leakage of endogenous serum immunoglobulin G (IgG) and a 70 kDa dextran-TMR administered intravenously at 48-hours post-t-MCAO. No significant differences in either the volume, area, or fluorescence intensity of serum IgG leakage were observed between the WT and *Rab7a*^*iECKO*^ brains (Figure S1**A-G**). However, we could not detect any 70 kDa dextran-TMR leakage in the ipsilateral cortex of both genotypes at 48 h after t-MCAO after extraction of the tracer from the brain and measurement of fluorescence intensity (Figure S1**H**), suggesting that the BBB was not leaky to 70 kDa dextran-TMR 48 h after t-MCAO. Thus, serum IgG leakage likely reflects BBB leakage at an earlier timepoint after t-MCAO. Caveolin-1 (Cav-1) protein mediates transcellular transport across ECs. We analyzed whether endothelial Rab7a elimination affected the levels of Cav-1 protein, and found that they were increased in total brain lysates from the ipsilateral cortex of *Rab7a*^*fl/fl*^ (WT) and *Rab7a*^*iECKO*^ mice 48 h after t-MCAO (Figure S1**I**,** J**). Thus, Rab7a EC-specific deletion did not affect the acute transcellular BBB leakage after ischemic stroke or the upregulation of Cav-1 protein. In summary, Rab7a EC-specific deletion rescues significantly the acute changes in paracellular, but not transcellular, BBB permeability after ischemic stroke.

### Rab7a^iECKO^ mice show improved neuronal outcomes 48 h after t-MCAO

**Fig. 3:**
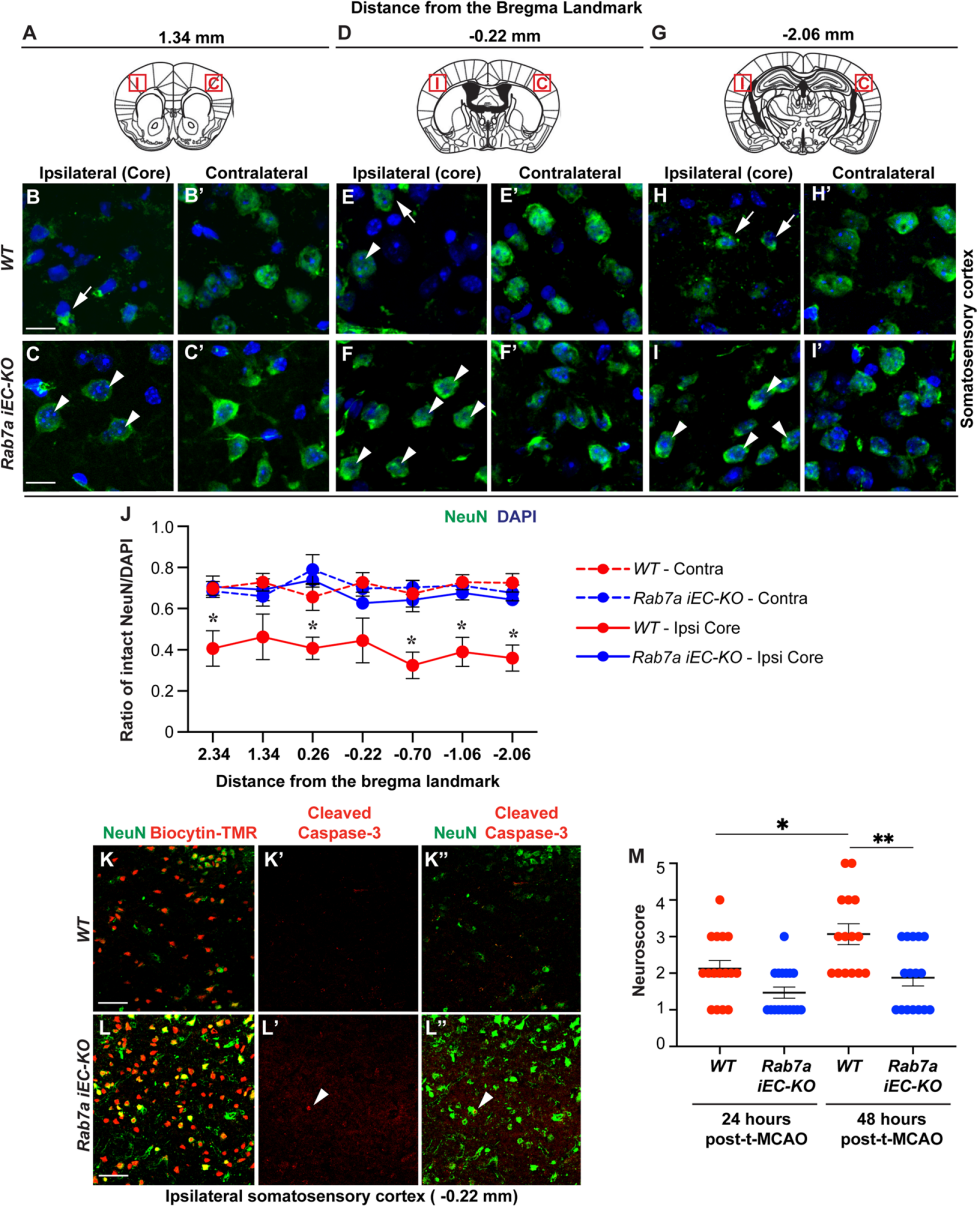
*Rab7a*^*iECKO*^ mice show improved neuronal survival and neurological score 48 h after t-MCAO. (**A-I’**) Immunofluorescence analysis for NeuN (green) and DAPI (blue) in the contralateral and ipsilateral cortex (core region) of WT (*Rab7a*^*fl/fl*^) and *Rab7a*^*iECKO*^ mice 48 hours after t-MCAO. (**A**,** D**,** G**) Diagrams illustrate the brain sections at the three distances from the bregma landmark (1.34, -0.22, -2.06) and the red boxes outline the cortical area shown in each micrograph in **B-I**’. (**B-I’**) White arrows indicate abnormal and white arrowheads indicate normal NeuN staining in the ipsilateral cortex (core region). Scale bar: 25 µm. (**J**) Quantification of the fraction of NeuN + neurons with normal staining over DAPI in the contralateral (dotted lines) and ipsilateral (core regions, solid lines) cortices (Y axis) in seven brain sections located at different distances from the bregma landmark (X axis) of WT and *Rab7a*^*iECKO*^ mice 48 hours after t-MCAO. Each dot represents the average of n = 8 animals / group. Data are means ± s.e.m. *: *p* < 0.05; one-way ANOVA with post-hoc Tukey’s correction. **(K-L”)** Immunofluorescence images for either biocytin-TMR (red) outlining the area of BBB leakage and NeuN (green), or cleaved Caspase-3 (red) and NeuN (green) in the core regions of the ipsilateral cortex of *WT* and *Rab7a*^*iECKO*^ mice at 48 h after t-MCAO. There is no expression of cleaved Caspase-3 by NeuN-positive surviving neurons in either genotype. Scale bar: 50 μm (**O**) Analysis of neurological score in WT and *Rab7a*^*iECKO*^ mice 24 and 48 h after t-MCAO. Each dot represents one animal (*n* = 14 WT and *n* = 16 *Rab7a*^*iECKO*^ mice from three independent cohorts). Data are medians ± 95% C.I. *: *p* < 0.05; **: *p* < 0.01; Kruskal-Wallis one-way ANOVA

Neurons in the hypoperfused, but not yet dead, peri-infarct region, or penumbra located in the cortex are threatened but can potentially be salvaged with reperfusion and when the BBB does not experience severe damage in the t-MCAO model (reviewed in [3]). To determine whether Rab7a-mediated rescue in acute paracellular BBB permeability affect neuronal health in the cortical reperfusion territory after t-MCAO, we assessed the quality and number of NeuN^+^ neurons in the ipsilateral and contralateral somatosensory cortices of both genotypes in brain sections at seven distinct distances from the bregma landmark (Fig. 3A). NeuN staining appeared absent or fragmented in the ipsilateral somatosensory cortex (core region) of *Rab7a*^*fl/fl*^ (WT) mice, but it appeared largely normal in the ipsilateral cortex (core region) of *Rab7a*^*iECKO*^ mice (Fig. 3B-I**’**), although both regions had extensive IgG leakage. Quantification of the ratio of NeuN^+^ cells with intact NeuN staining / DAPI^+^ cells showed a significant 2-fold reduction in the ipsilateral compared to the contralateral cortex of WT mice (Fig. 3J). However, the ratio of NeuN^+^ cells with intact NeuN staining / DAPI^+^ cells was comparable between the ipsilateral and contralateral cortexes of *Rab7a*^*iECKO*^ mice in all brain sections (Fig. 3J). We could not detect any cleaved-Caspase3^+^ apoptotic neurons in the ipsilateral cortex at 48 hours post-t-MCAO regardless of the genotype (Fig. 3K-L**”**). Consistent with a greater number of normal-appearing NeuN^+^ neurons in the ipsilateral cortex, *Rab7a*^*iECKO*^ showed fewer neurological deficits with a lower modified Bederson neurological score (0–5) using a series of behavioral tests (forelimb flexion, circling, resistance to lateral push as published [47]), compared to WT mice by 48 h after t-MCAO (Fig. 3M). Therefore, ablation of endothelial Rab7 rescues acute BBB disruption and reduces neuronal injury/death in the cortical regions at 48 h post-t-MCAO.

**Fig. 4:**
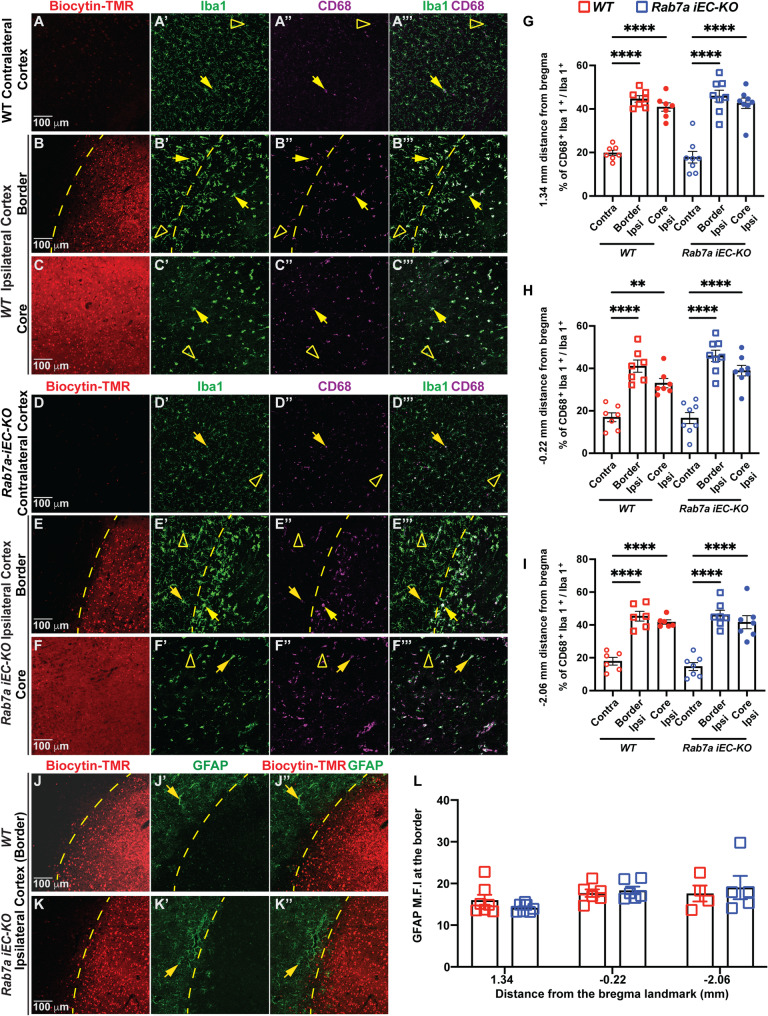
*Rab7a*-mediated rescue of BBB permeability after t-MCAO is not associated with reduced microglia activation / macrophage infiltration or astrocyte activation. **(A-F’’’)** Immunofluorescences images for biocytin-TMR (red), Iba1 (green), and CD68 (magenta) in either the contralateral cortex **(A-A’’’**,**D-D’’’)**, or border **(B-B’’’**,** E-E’’’)**, and core **(C-C’’’**,**F-F’’’)** regions of the ipsilateral cortex of *Rab7a*^*fl/fl*^*(WT)* and *Rab7a*^*iECKO*^ mice 48 h after t-MCAO. Yellow arrowheads point to Iba1^+^, CD68^+^ activated myeloid cells, open yellow arrowhead indicate Iba1^+^ resting microglia. Scale bar = 100 μm. Quantification of activated / resting myeloid cells at three distances from the bregma landmark: **(G)** 1.34, **(H)** -0.22, and **(I)** -2.06 in either the contralateral cortex, or border and core regions of the ipsilateral cortex of *WT* and *Rab7a*^*iECKO*^ mice 48 h after t-MCAO. Each dot represents an animal (*n* = 6–9 mice/group). Data are means ± s.e.m. ***: *p* < 0.001; **: *p* < 0.01; *: *p* < 0.05; one-way ANOVA with post-hoc Tukey’s correction. **(J-K’’)** Immunofluorescences images for biocytin-TMR (red) and GFAP (green) in the border of the ipsilateral cortex of *WT* and *Rab7a*^*iECKO*^ mice 48 h after t-MCAO. Yellow arrowheads point to GFAP^+^ reactive astrocytes at the stroke border. Scale bar = 100 μm. **(L)** Quantification of GFAP mean florescence intensity (M.F.I) at the stroke border in brain sections at three distances from the bregma landmark 1.34, -0.22, -2.06 in *WT* and *Rab7a*^*iECKO*^ mice 48 h after t-MCAO. Data are means ± s.e.m and normalized to WT mice. ***: *p* < 0.001; **: *p* < 0.01; *: *p* < 0.05; one-way ANOVA with post-hoc Tukey’s correction

To examine if the reduced paracellular BBB permeability found in *Rab7a*^*iECKO*^ mice after ischemic stroke is accompanied by reduced inflammatory responses of glial cells, we analyzed the percentage of activated myeloid cells (activated microglia and infiltrating macrophages) and resting microglia in the ipsilateral (core and border regions) and contralateral cortex of both genotypes 48 hours after t-MCAO in brain sections at three distinct distances from the bregma landmark with significant rescue of BBB damage (Fig. 4A-I). Resting myeloid cells (mostly Iba1^+^ CD68^−^ microglial cells with ramified branches) were found outside the BBB leakage area in the infarct border region of the ipsilateral cortex and contralateral cortex (Fig. 4A-B**’”**,** D-E’”**; open arrowheads). In contrast, activated myeloid cells (Iba1^+^ CD68^+^ cells with ameboid morphology) were found in the core and border regions of the ipsilateral cortex in both WT and *Rab7a*^*iECKO*^ cortexes (Fig. 4B-C**’”**,** E-F’”**; yellow arrows). Although the ratio of CD68^+^ Iba1^+^ over total Iba1^+^ cells was higher in the core and border regions in the ipsilateral cortex compared to the contralateral cortex, there were no significant differences between the two genotypes (Fig. 4G-I). In addition, there was no difference in the mean fluorescence intensity of GFAP reactivity, a marker of activated astrocytes, in the border region of the ipsilateral cortex (reperfusion territory) in both genotypes at 48 h post-t-MCAO (Fig. 4J-L). The comparable inflammatory responses of both myeloid cells and astrocytes between WT and *Rab7a*^*iECKO*^ cortexes in the acute phase (48 h) of ischemic stroke could reflect the partial, rather than a complete, rescue in BBB permeability observed in *Rab7a*^*iECKO*^ mice since there is extensive IgG leakage.

### Endothelial Rab7a elimination reduces structural abnormalities at the BBB tight junctions 48 h after t-MCAO

**Fig. 5:**
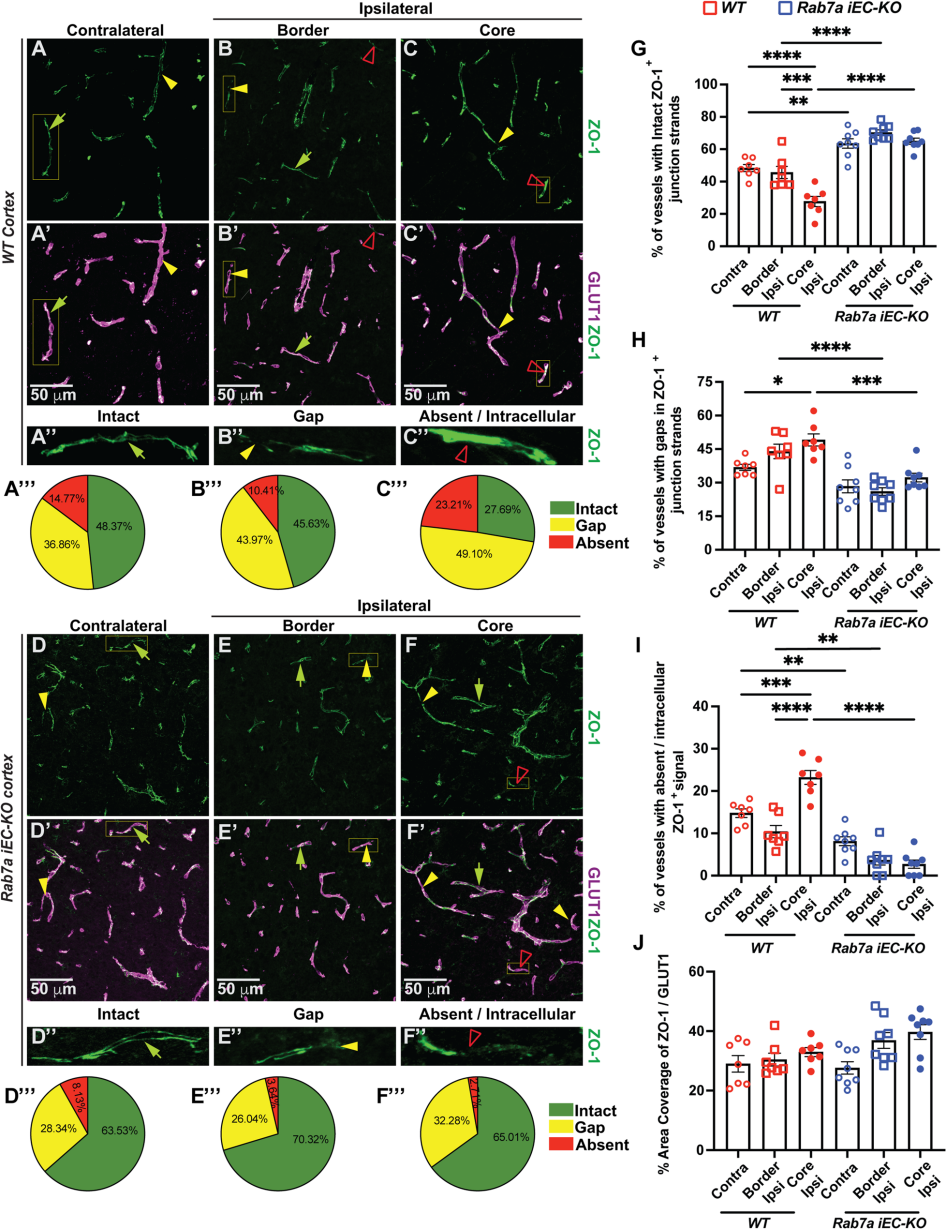
Endothelial *Rab7a* elimination reduces ZO-1 structural abnormalities in tight junctions 48 h after t-MCAO. (**A-F”’**) Immunofluorescence images for ZO-1 (green) and Glut1 (magenta) in either the contralateral cortex **(A-A’**,**D-D’)**, or border **(B-B’**,**E-E’)** and core **(C-C’**,**F-F’)** regions of the ipsilateral cortex of *WT* and *Rab7a*^*iECKO*^ mice 48 h after t-MCAO. Normal green arrowhead indicates intact ZO-1^+^ tight junction (TJ) strands, yellow arrowhead points towards gaps in ZO-1^+^ TJ strands, red open arrowheads indicate absent ZO-1 + TJ strands. Scale bar = 50 μm. **(A’’-F’’)** Magnified images of the white boxed areas in **(A-F’)** to illustrate the phenotype of the ZO-1^+^ TJ strand. **(A”’- F”’)** Pie charts correspond to the percentage of intact (green), gap (yellow), and absent (red) ZO-1^+^ junctional strands in the contralateral and ipsilateral cortex (core and border) of *WT* and *Rab7a*^*iECKO*^ mice 48 h after t-MCAO. **(G-I)** Dotted bar graphs of the percentage of vessel segments with (**G**) intact TJ segments, **(H)** TJ strands with gaps, **(I)** absent ZO-1 in either the contralateral cortex, or border and core regions of the ipsilateral cortex of *WT* and *Rab7a*^*iECKO*^ mice 48 h after t-MCAO. **(J)** Dotted bar graph of the percentage of the vascular area covered by ZO-1^+^ TJ strands. Each dot represents an animal (*n* = 7/8 mice/group). Data are means ± s.e.m. ***: *p* < 0.001; **: *p* < 0.01; *: *p* < 0.05; one-way ANOVA with post-hoc Tukey’s correction

To understand how Rab7a affects the disassembly of BBB tight and adherens junctions during the acute phase of ischemic stroke, we analyzed the morphology of eGFP::Claudin-5^+^, ZO-1^+^ TJs and CDH5^+^ AJs at 48 hours after t-MCAO by immunofluorescence and electron microscopy. We first quantified the fraction of Glut1^+^ vessel segments with either intact eGFP^+^ junction strands, junction strands with large gaps (> 2.5 µm), or absent junctions from 6–8 independent regions of interest (ROIs) located at either the core and border areas of the ipsilateral cortexes or contralateral cortexes (Figures S2A-C”, S2D-F”) of two brain Sect. (0.26 to -0.22 from the bregma landmark) with the highest rescue in acute BBB leakage (Fig. 2F). In the last category, we also included vessel segments where eGFP::Claudin-5^+^ protein was located throughout the Glut-1 + positive BECs (intracellular localization; Figure S2F”). The majority (~ 63–66%) of eGFP::Claudin-5^+^ TJs strands were intact in the contralateral cortexes regardless of the genotype, and this fraction was significantly decreased (~ 30.6%) in the core region of the ipsilateral cortexes of *Rab7a*^*fl/fl*^ (WT) mice at 48 hours after t-MCAO (Figure S2A-A”’,** G**). In contrast, there was a significantly higher fraction of vessel segments with TJ strand gaps [~ 50%; Figure S2B-B”’,** H**] and absent junctions or intracellular GFP^+^ inclusions [~ 20%; Figure S2C-C”’,** I**, Movie S3] in the core, compared to either the border of ipsilateral cortexes or contralateral cortexes of WT mice. Although, the fraction of vessel segments with TJ strand gaps was reduced in the core of *Rab7a*^*iECKO*^ ipsilateral cortexes (~ 40%), and there was no longer a significant difference among the core and border ROIs in the ipsilateral cortex with the contralateral cortexes in *Rab7a*^*iECKO*^ mice for this parameter (TJ strand gaps), we could not detect statistical significant differences either in the fraction of vessel segments with TJ strand abnormalities (Figure S2E-F”’,** H**,** J**, Movie S4), or vessels area covered by intracellular eGFP^+^ signal (Figure S2J) between two genotypes. This finding likely reflects the large variability within the ipsilateral cortical ROIs of mice with the same genotype inherent in t-MCAO. VE-Cadherin^+^ (CDH5^+^) AJs showed a similar behavior to eGFP::Claudin-5^+^ TJs (90–95% correlation) at the BBB after ischemic stroke with no significant differences observed between two genotypes (Figure S3). In contrast, *Rab7a*^*iECKO*^ mice had a reduced fraction of vessel segments with ZO-1^+^ TJ strands containing gaps (~ 32%) or no ZO-1 signal (4%) in the core ROIs of the ipsilateral cortex compared to WT mice [ZO-1^+^ TJ strand with gaps (~ 49%) and absent TJs (23.2%); Fig. 5A-I, Movie S1, S2]. Moreover, *Rab7a*^*iECKO*^ mice had a significant higher fraction (65%) of vessels segments with intact ZO-1^+^ TJ strands compared to WT mice (~ 27.5%) in core ROIs of the ipsilateral cortex at 48 h post-t-MCAO (Fig. 5A-A**”’**,** D-D”’**,** G**). Overall, these data show that Rab7a elimination in BECs predominantly reduces ZO-1 degradation and localization to cell junctions compared to Claudin-5 or VE-Cadherin during the acute BBB damage after ischemic stroke.

**Fig. 6:**
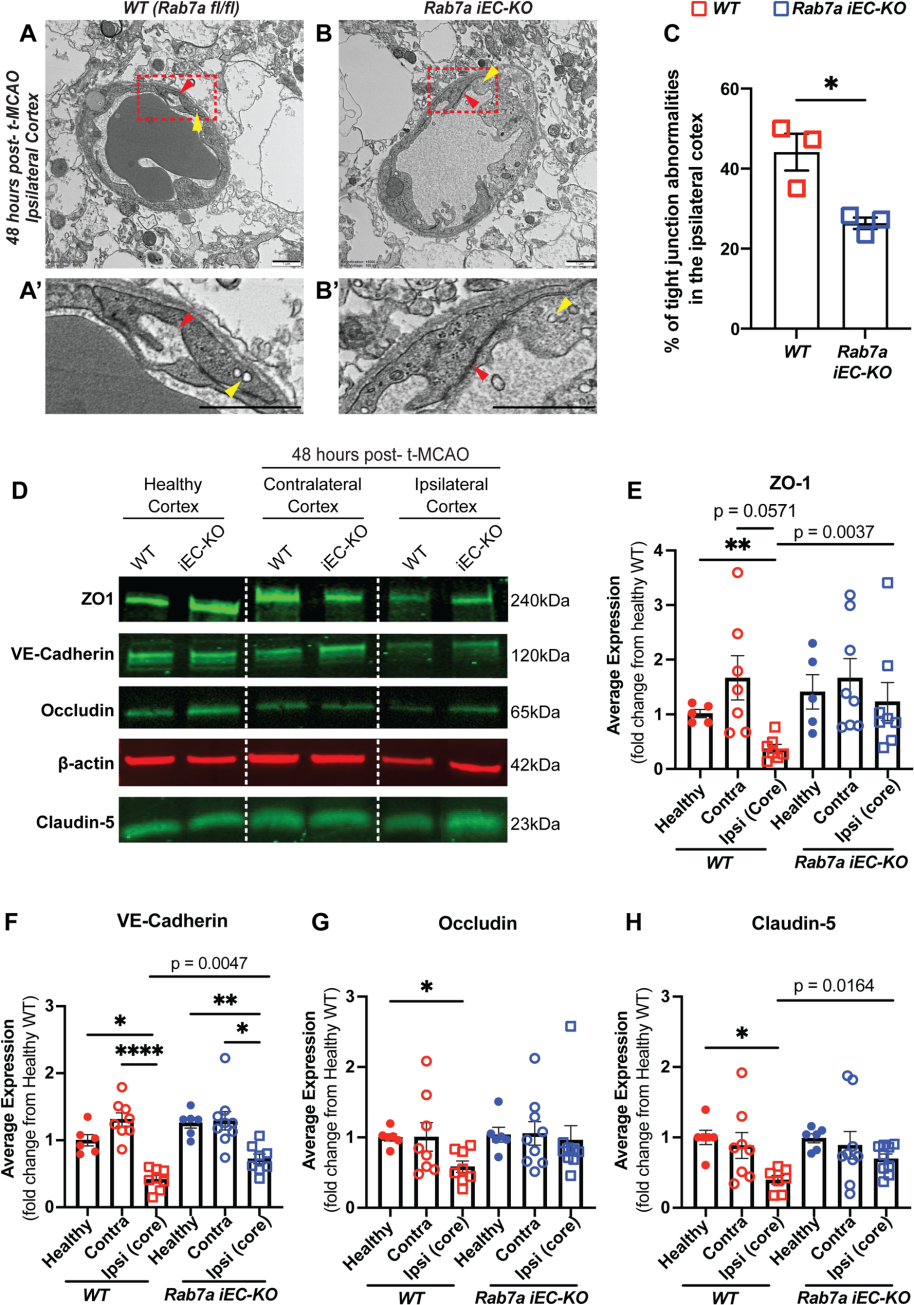
*Rab7a* regulates degradation of select BBB junctional proteins 48 h after t-MCAO. **(A-B’)** Transmission Electron Microscopy (TEM) images of tight junctions 48 h post-t-MCAO in the ipsilateral cortex (core region) of *Rab7a*^*fl/fl*^ (WT) and *Rab7a*^*iECKO*^ mice. Red arrowheads point to tight junctions, yellow arrowheads point to caveolae. **(A’-B’)** Magnified images of boxed areas (**A**,** B**) to illustrate normal and abnormal tight junctions in brain endothelial cells. Scale bar = 1 μm. **(C)** Quantification of tight junction abnormalities in the ipsilateral cortex of WT and *Rab7a*^*iECKO*^ mice. The data were analyzed from 46–55 tight junctions per mouse obtained from 16–22 images in the ipsilateral cortical (core) region (*n* = 3 mice / genotype). Data are means ± s.e.m. *: *p* < 0.05; Mann-Whitney t-test. (**D**) Western blots for BBB junctional proteins ZO-1, VE-cadherin, Occludin and Claudin-5 and β-actin (control) of tissue lysates collected from either the healthy, contralateral and ipsilateral (core) ischemic cortex of *WT* and *Rab7a*^*iECKO*^ mice 48 h after t-MCAO. The respective molecular weights for each protein are shown on the right. (**E-H**) Quantification of Claudin-5, Occludin, ZO-1 and VE-cadherin protein levels in either healthy, contralateral or ipsilateral ischemic cortical lysates of *WT* and *Rab7a*^*iECKO*^ mice 48 h after t-MCAO. Each dot represents an animal (6–9 mice / group). Data are means ± s.e.m and normalized to protein levels in WT healthy mice. ***: *p* < 0.001; **: *p* < 0.01; *: *p* < 0.05; Brown-Forsythe and Welch ANOVA. The shown *p* values are comparisons between the ipsilateral ischemic cortical lysates of *WT* and *Rab7a*^*iECKO*^ mice 48 h after t-MCAO were achieved by Mann-Whitney t-test

Next, we examined the ultrastructural morphology of endothelial TJs in the ipsilateral core cortices of WT and *Rab7a*^*iECKO*^ mice at 48 hours after t-MCAO with transmission electron microscopy. We acquired 16–22 images in the core ROIs of the ipsilateral cortex and analyzed 46–55 endothelial TJs per animal. At 48 hours after t-MCAO, on average 45% of BBB TJs in the core ROIs of *Rab7a*^*fl/f*l^ (WT) ipsilateral cortex had severe structural abnormalities containing large gaps where adjacent membranes were separated from each other due to loss of the electron dense proteinaceous material (Fig. 6A, A**’**,** red arrowhead**,** C**), consistent with our prior findings in ischemic stroke [11]. In contrast, the fraction of TJ with these structural abnormalities was significantly reduced (~ 25%) in the core ROIs of *Rab7a*^*iECKO*^ ipsilateral cortex at 48 h after t-MCAO (Fig. 6B, B**’**,** C**). Thus, Rab7a elimination in BECs reduces acute structural abnormalities of BBB TJs after ischemic stroke also at the electron microscopy level.

To determine the effects of endothelial Rab7a elimination on AJ (VE-Cadherin) and TJ [Claudin-5, Occludin, and ZO-1] protein levels in BECs after ischemic stroke, lysates were collected from either the ipsilateral (core region) and contralateral cortices of both genotypes at 48 h after t-MCAO and assessed by Western blotting (Fig. 6D). VE-Cadherin, Claudin-5, Occludin and ZO-1 protein levels were significantly reduced by approximately 2-fold in the ipsilateral cortex of WT mice at 48 h after t-MCAO, compared to both the WT healthy (t-MCAO sham) and WT contralateral cortex (Fig. 6E-H). Similarly, VE-Cadherin protein was significantly reduced in *Rab7a*^*iECKO*^ ipsilateral cortex compared to either the *Rab7a*^*iECKO*^ healthy or contralateral cortex (Fig. 6F). In contrast, there was no significant difference in Claudin-5, Occludin or ZO-1 in *Rab7a*^*iECKO*^ ipsilateral cortex compared to either the *Rab7a*^*iECKO*^ healthy or contralateral cortex when we performed a group analysis (Brown-Forsythe Welsh ANOVA) [ZO1 (*p* = 0.3479), VE-Cadherin (*p* = 0.0694), and Claudin-5 (*p* = 0.0717)] likely due to smaller effect size, the larger variability within the same genotype and smaller number of mice (data not shown). However, when we compare the AJ and TJ protein levels between the ipsilateral cortexes of the two genotypes using a Mann-Whitney t-test, there is a significant increase in ZO1 (*p* = 0.0037), VE-Cadherin (*p* = 0.0047), and Claudin-5 (*p* = 0.0164) levels in the ipsilateral *Rab7*^*iECKO*^ cortex compared to WT cortex (Fig. 6E, G, H). These data support the hypothesis that Rab7a elimination in BECs can partially rescue the degradation of select AJ and TJ proteins after ischemic stroke, although the effects are not very robust.

### Pro-inflammatory cytokines, TNFα and IL1β, but not glucose and oxygen deprivation, induce Rab7a activation in BECs in vitro

**Fig. 7:**
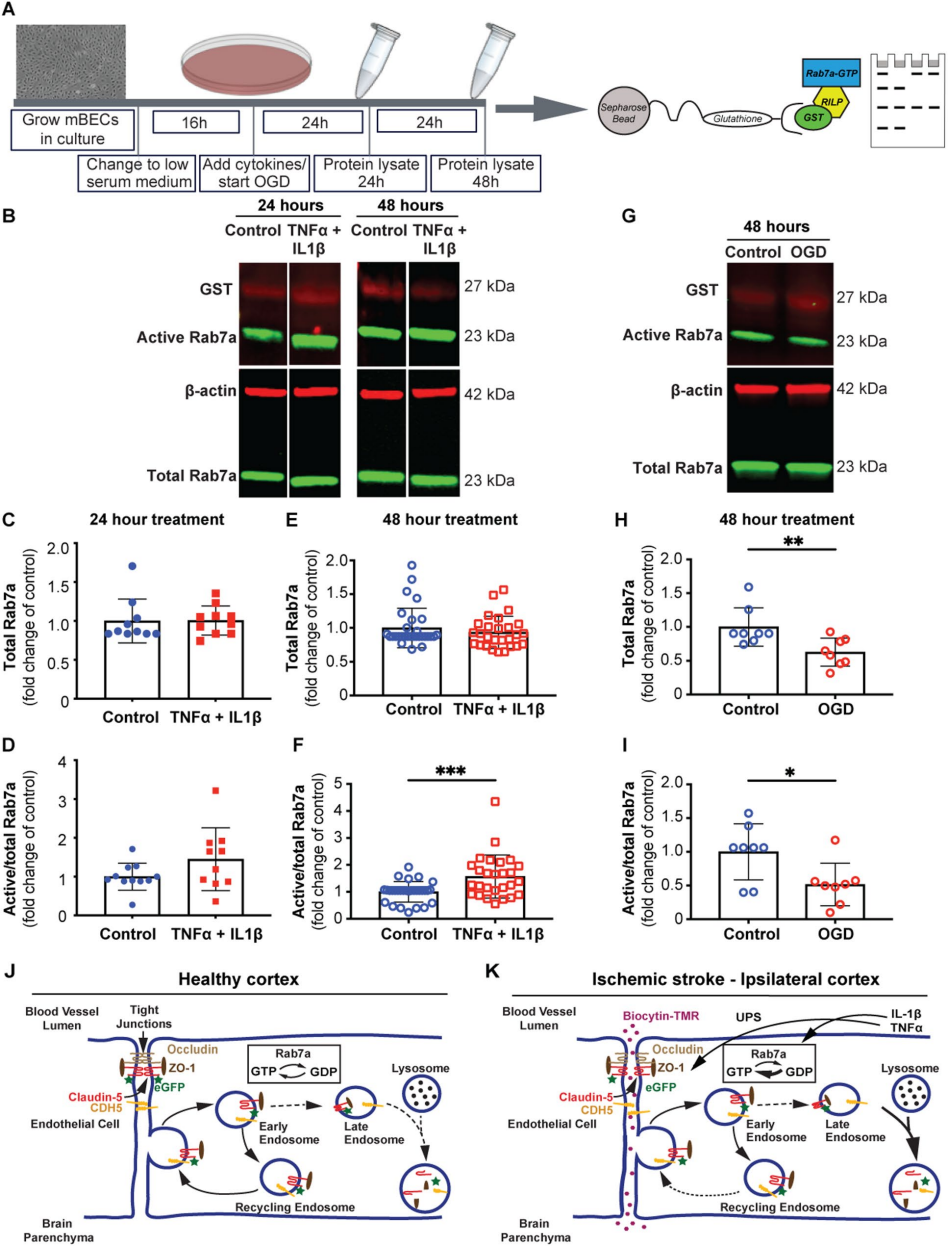
Proinflammatory cytokines TNFα and IL1β induce Rab7a activation in mouse brain endothelial cells. (**A**) Schematic diagram of the experimental setup. Primary mouse brain endothelial cells (mBECs) are grown to confluence and switched to low-serum media prior to addition of TNFα, IL-1β (10 ng/mL). Protein lysates are collected after 24–48 h from the start of cytokine treatment. Rab7a-GTP is pulled down using a GST-RILP fusion protein that binds glutathione immobilized on sepharose beads. Western blotting detects Rab7a and Rab7a-GTP protein levels. (**B**) Western blot of active and total Rab7a proteins in mBECs treated with TNFα and IL1β for either 24–48 h and untreated control cells. GST and β-actin are used to normalize active Rab7a and total Rab7a levels in the eluate or protein lysate, respectively. The molecular weight of each protein is shown on the right. (**C-F**) Quantification of total (**C**,** E**) and active (**D**,** F**) Rab7a levels in mBECs treated with TNFα and IL1β for 24 h (**C**,** D**) and 48 h (**E**,** F**) and untreated cells. Each dot represents an independent experiment. Data are means ± s.e.m. (**F**) ***: *p* < 0.005; Student’s t-test. (**G**) Western blot for active and total Rab7a in mBECs grown in oxygen and glucose deprivation (OGD) conditions for 48 h and untreated control cells. The molecular weight of each protein is shown on the right. (**H**,** I**) Quantification of total (**H**) and active (**I**) Rab7a levels in mBECs grown in OGD conditions for 48 h and control cells. Each dot represents an independent experiment. Data are means ± s.e.m.; **: *p* < 0.01; *: *p* < 0.05; n.s.: *p* > 0.05 (not shown) Student’s t-test. (**J**, **K**) Model for the role of Rab7a in regulation of select BBB junctional proteins after ischemic stroke. In healthy conditions, Rab7a balances the degradation and recycling of internalized adherens (VE-Cadherin, CDH5, yellow) and tight junction [Claudin-5 (red) and ZO-1 (brown)] proteins. In ischemic stroke, some inflammatory cytokines (e.g. IL-1β, TNFα) activate Rab7a to increase degradation of select junctional proteins in BECs leading to acute BBB disruption. Rab7a-independent mechanisms also contribute to degradation of BBB junctional proteins at the acute phase of ischemic stroke

Rab7a cycles between inactive (GDP-bound) and active (GTP-bound) states to regulate various cell biological processes [28, 29]. Rab-interacting lysosomal protein (RILP) binds selectively to Rab7-GTP and recruits the dynein-dynactin motor complex to facilitate vesicle movement toward the minus end of microtubules [50]. The N terminus region of RILP is required to recruit dynein motors, but not for Rab7-GTP binding; therefore an N-terminal truncation of RILP can be used to assess Rab7 activation in cells [31, 33]. To investigate which cytokines may induce Rab7a activation in ischemic stroke, we cultured primary mouse BECs in vitro under conditions of either oxygen and glucose deprivation (OGD), or inflammation (TNFα and IL-1β; 10 ng/mL) for 48 h and quantified the amount of GTP-bound Rab7a by using a GST-RILP fusion protein to pull down selectively the active protein from cell lysates as described [31, 33] (Fig. 7A; see Methods for more details). Both the total and active Rab7a levels were decreased significantly after 48 h of OGD treatment (Fig. 7G-I), suggesting that ischemic conditions do not activate Rab7a protein. Since ischemic stroke induces inflammation which exacerbates BBB disruption [14], we then tested whether distinct pro-inflammatory cytokines induce Rab7a activation. Treatment with Tumor Necrosis Factor α (TNFα) and Interleukin 1β (IL1β) did not increase the total amount of Rab7a, but increased, albeit non-significantly, the GTP-bound Rab7a levels after 24 h (Fig. 7B-D). After 48 h of TNFα and IL1β treatment, Rab7a-GTP levels, but not total Rab7a levels, were significantly increased (Fig. 7B, E, F). Three other pro-inflammatory cytokines that are upregulated after ischemic stroke, IL-21 [51], CCL2 [52] and IL-17 A [53], had no effect on either total or activated Rab7a levels in BECs (Figure S4). These findings suggest that select pro-inflammatory cytokines promote Rab7a activation in primary BECs in vitro, and likely in the brain vasculature, to promote degradation of BBB AJ and TJ proteins during the acute phase of ischemic stroke.

## Discussion

The initial upregulation of endothelial transcytosis contributes together with delayed disassembly of cell junctions due to persistent degradation of adherens and tight junction proteins to increase BBB permeability after ischemic stroke (reviewed in [3]). However, the cell biological mechanisms driving junctional disassembly are not fully understood. OGD has been proposed to promote junctional protein degradation through activation of several MMPs that cleave the extracellular domains of select AJ and TJ proteins in BECs [54, 55]. Surprisingly, OGD does not activate Rab7a in primary BECs, suggesting that OGD-induced junctional protein degradation does not require Rab7a. On the contrary, IL-1β and TNF-α, which are upregulated in stroke tissue between 24 and 48 h after reperfusion and coincide with immune cell infiltration into the CNS (reviewed in [13, 14]), induce internalization and degradation of select BBB AJ and TJ proteins [22, 26]. One of the mechanisms by which IL-1β and TNF-α mediate BBB cell junction protein degradation is via Rab7a activation, which then degrades select BBB cell junction-associated proteins. Other pro-inflammatory cytokines/chemokines that have been implicated in BBB dysfunction such as IL-17 A, IL-21 and CCL2 may promote TJ degradation through Rab7a-independent mechanisms, since they cannot activate Rab7a in primary BECs. Therefore, pro-inflammatory cytokines likely mediate BBB TJ degradation at the acute phase of ischemic stroke through both Rab7a-dependent and -independent mechanisms.

Cav-1 has been previously implicated in tight junction transmembrane protein internalization and degradation primarily from in vitro studies [22, 26, 54]. Our data demonstrate that Rab7a-mediated rescue of acute BBB dysfunction after stroke in vivo is not accompanied by changes in Caveolin-1 protein levels or transcellular permeability in vivo, although the inflammatory milieu in stroke increases Cav-1 protein levels in BECs. Although we cannot exclude the possibility that Caveolin-1-dependent mechanisms degrade select BBB junctional proteins under some conditions, it is likely not the main mechanism by which IL-1β and TNFα mediate degradation of cell junction proteins in acute ischemic stroke. Future studies using endothelial-specific ablation of IL-1R1/TNF receptors combined with endothelial-targeted Rab7a knockdown/rescue during the inflammatory challenge will provide clearer insights into the mechanisms by which these pro-inflammatory cytokines act to degrade BBB structure and function after ischemic stroke and guide therapeutic interventions. In summary, the cellular mechanisms of BBB AJ and TJ protein degradation are context dependent and likely differ across distinct neurological diseases and stages of disease.

Overall our findings from in vivo studies in mice suggest a model for how the state of Rab7a activation in BECs regulates, in part, degradation of select BBB junctional proteins in healthy and disease states (Fig. 7J, K). We propose that under physiological conditions, similar levels of active and inactive Rab7a protein levels maintain a constant internalization, recycling and degradation of adherens and tight junction proteins, in order to retain constant levels at cell junctions (Fig. 7J). When BECs are exposed to a subset of pro-inflammatory cytokines (e.g. TNFα and IL1β), such as in acute ischemic stroke, Rab7a is activated. Rab7a may be activated by multiple mechanisms through a GEF such as Ccz1 [35–37], reducing TBC1D15 (GAP protein) levels as shown in vitro [49, 56] and in a mouse model of myocardial infarction [57], or through post-translational protein modifications (reviewed in [58]). Rab7a activation pushes the endolysosomal pathway towards degradation, rather than recycling, and drives cell junctional proteins toward the lysosomes which further exacerbates acute changes in paracellular BBB permeability after ischemic stroke (Fig. 7K). Our in vivo data suggest that endothelial Rab7 elimination is more effective in reducing abnormal ZO-1 localization at BBB cell junctions after ischemic stroke compared to eGFP::Claudin-5 and VE-Cadherin proteins and preserving their total protein levels. In rodent stroke models, hypoxia-inducible factor 1 (HIF-1) and VEGF-A are also rapidly induced within 1 h after ischemia [59, 60]. VEGF-A expression correlates temporally with BEC proliferation in the peri-infarct region [60–62]. Post-ischemic VEGF-A administration also increases BBB leakage and hemorrhagic transformation, while antagonism of endogenous VEGF-A reduces infarct volume [63, 64]. We cannot exclude the possibility that endothelial Rab7a deletion may affect VEGFR2 receptor trafficking in BECs and their responses to VEGF-A after ischemic stroke; however, the major effect of Rab7a elimination is directly through regulation of BBB adherens and tight junction protein trafficking in response to the inflammatory milieu found in ischemic stroke.

The mechanism by which Rab7a activation triggers degradation of BBB junctional proteins to promote disassembly of BEC cell junctions after ischemic stroke implies a potential lack of selectivity, since the endolysosomal degradation is a fundamental cell biological process. The genetic loss-of-function data suggest that Rab7a knockdown has no effect on the levels of Caveolin-1 which is an integral membrane protein. In contrast, Rab7a elimination in vivo rescues the levels and localization of select BBB junctional proteins such as ZO-1 and to a lesser extent Claudin-5 and VE-Cadherin in vivo. However, it has very little effect on Occludin which is a member of the MARVEL family of transmembrane proteins. Moreover, Rab7a elimination rescues only partially the degradation of ZO-1, Claudin-5 and VE-Cadherin proteins, suggesting that Rab7a-dependent and Rab7a-independent mechanisms may degrade BBB junctional proteins under inflammatory conditions. Inflammatory cytokines also promote ubiquitin-mediated proteasome degradation of VE-cadherin [65], Claudin-5 [66] and ZO-1 (reviewed in [20]). Rab7a activation is also critical to promote autophagy which together with the ubiquitin-proteasome system serve as two independent mechanisms to degrade and clear protein debris under cellular stress responses such as ischemic stroke (reviewed in [67]). It is conceivable that both mechanisms are activated by inflammatory cytokines in BECs after ischemic stroke to degrade damaged AJ and TJ proteins and promote formation of new junctions at the BBB for post-stroke recovery.

The major effects of Rab7a elimination on preservation of BBB structure and functions were seen in the cortical regions which contains salvageable tissue whose fate is modifiable by multiple mechanisms including endothelial-mediated mechanisms related to BBB function. This is where BBB leakage, edema, and secondary inflammation drive neuronal injury, and where an endothelial Rab7a-mediated effect on the BBB function would be expected to manifest for neuronal survival. Consistent with this rationale, we found preserved neuronal morphology in the ipsilateral cortical regions of *Rab7a*^*iECKO*^ mice along with improved neurological scores compared to WT mice. These findings link endothelial cell Rab7a activation with the acute BBB integrity after reperfusion and neuronal outcomes in the tissue compartment where such a link is mechanistically testable, namely the cortical penumbra, which becomes infarcted with the infarct growth in wild-type mice, but not completely in *Rab7a*^*iECKO*^ mice.

Overall, our study identifies a new molecular mechanism controlling BEC junctional integrity in diseased BBB, and establishes Rab7a as an important regulator of AJ and TJ protein turnover via the endolysosomal pathway under inflammatory conditions. Our findings have implications not only for ischemic stroke, but also other neurological disorders characterized by inflammation and BBB dysfunction. For example, in EAE, structural abnormalities in BBB TJs precede the onset of disease and persist throughout the course of EAE to allow immune cell infiltration into the CNS [12], emphasizing a critical role for pro-inflammatory mechanisms in driving TJ strand disassembly. Based on our findings, we predict that early and persistent Rab7a activation due to pro-inflammatory cytokines Il-1β and TNF-α may likely drive degradation of BBB TJ transmembrane proteins in EAE leading to BBB dysfunction [12].

## Conclusion

In conclusion, our findings provide evidence for a new mechanism by which inflammatory cytokines promote degradation of select junctional proteins via Rab7a activation, leading to BBB dysfunction after ischemic stroke.

## Supplementary Information

Below is the link to the electronic supplementary material.

Supplementary Material 1

## Data Availability

All data are available in the manuscript and the supplementary information. There are no codes used in this study. Any additional information required to reanalyze the data reported in this study will be available upon request.

## Abbreviations

AJs: Adherens junctions
BECs: Brain endothelial cells
Biocytin-TMR: Biocytin-tetramethylrhodamine
BBB: Blood-brain barrier
Cldn-5: Claudin-5
Cav-1: Caveolin-1
CNS: Central Nervous System
EEA-1: Early Endosomal Antigen
GFP: Green Fluorescent Protein
GEF: Guanidine Exchange Factor
IL1β: Interleukin 1β
LBK1: Liver kinase B1
MMPs: Matrix metalloproteases
OGD: Oxygen and glucose deprivation
t-MCAO: Transient middle cerebral artery occlusion
TNFα: Tumor necrosis factor
TJs: Tight junction
ZO-1: Zonula Occludens 1

## References

[1] Biswas S, Cottarelli A, Agalliu D (2020) Neuronal and glial regulation of CNS angiogenesis and barriergenesis. Development 147(9) 10.1242/dev.182279 PMC7197727 32358096

[2] Collaborators GBDS (2021) Global, regional, and National burden of stroke and its risk factors, 1990–2019: a systematic analysis for the global burden of disease study 2019. Lancet Neurol 20(10):795–820 34487721 10.1016/S1474-4422(21)00252-0 PMC8443449

[3] Liebner S, Dijkhuizen RM, Reiss Y, Plate KH, Agalliu D, Constantin G (2018) Functional morphology of the blood-brain barrier in health and disease. Acta Neuropathol 135(3):311–336 29411111 10.1007/s00401-018-1815-1 PMC6781630

[4] Krupinski J, Kaluza J, Kumar P, Kumar S, Wang JM (1994) Role of angiogenesis in patients with cerebral ischemic stroke. Stroke 25(9):1794–1798 7521076 10.1161/01.str.25.9.1794

[5] Kanazawa M, Takahashi T, Ishikawa M, Onodera O, Shimohata T (2019) Del zoppo, angiogenesis in the ischemic core: A potential treatment target? J Cereb Blood Flow Metab 39(5):753–769 30841779 10.1177/0271678X19834158 PMC6501515

[6] Rust R (2020) Insights into the dual role of angiogenesis following stroke. J Cereb Blood Flow Metab 40(6):1167–1171 32065073 10.1177/0271678X20906815 PMC7238380

[7] Kaur C, Ling EA (2008) Blood brain barrier in hypoxic-ischemic conditions. Curr Neurovasc Res 5(1):71–81 18289024 10.2174/156720208783565645

[8] Sandoval KE, Witt KA (2008) Blood-brain barrier tight junction permeability and ischemic stroke. Neurobiol Dis 32(2):200–219 18790057 10.1016/j.nbd.2008.08.005

[9] Arai K, Jin G, Navaratna D, Lo EH (2009) Brain angiogenesis in developmental and pathological processes: neurovascular injury and angiogenic recovery after stroke. FEBS J 276(17):4644–4652 19664070 10.1111/j.1742-4658.2009.07176.x PMC3712842

[10] Hawkins BT, Davis TP (2005) The blood-brain barrier/neurovascular unit in health and disease. Pharmacol Rev 57(2):173–185 15914466 10.1124/pr.57.2.4

[11] Knowland D, Arac A, Sekiguchi KJ, Hsu M, Lutz SE, Perrino J, Steinberg GK, Barres BA, Nimmerjahn A, Agalliu D (2014) Stepwise recruitment of transcellular and paracellular pathways underlies blood-brain barrier breakdown in stroke. Neuron 82(3):603–617 24746419 10.1016/j.neuron.2014.03.003 PMC4016169

[12] Lutz SE, Smith JR, Kim DH, Olson CVL, Ellefsen K, Bates JM, Gandhi SP, Agalliu D (2017) Caveolin1 is required for Th1 cell infiltration, but not tight junction remodeling, at the Blood-Brain barrier in autoimmune neuroinflammation. Cell Rep 21(8):2104–2117 29166603 10.1016/j.celrep.2017.10.094 PMC5728697

[13] Doll DN, Barr TL, Simpkins JW (2014) Cytokines: their role in stroke and potential use as biomarkers and therapeutic targets. Aging Dis 5(5):294–306 25276489 10.14336/AD.2014.0500294 PMC4173796

[14] Rayasam A, Hsu M, Kijak JA, Kissel L, Hernandez G, Sandor M, Fabry Z (2018) Immune responses in stroke: how the immune system contributes to damage and healing after stroke and how this knowledge could be translated to better cures? Immunology 154(3):363–376 29494762 10.1111/imm.12918 PMC6002204

[15] Iadecola C, Buckwalter MS, Anrather J (2020) Immune responses to stroke: mechanisms, modulation, and therapeutic potential. J Clin Invest 130(6):2777–2788 32391806 10.1172/JCI135530 PMC7260029

[16] Doyle KP, Buckwalter MS (2020) Immunological mechanisms in poststroke dementia. Curr Opin Neurol 33(1):30–36 31789707 10.1097/WCO.0000000000000783 PMC7251986

[17] Endres M, Moro MA, Nolte CH, Dames C, Buckwalter MS, Meisel A (2022) Immune pathways in etiology, acute phase, and chronic sequelae of ischemic stroke. Circ Res 130(8):1167–1186 35420915 10.1161/CIRCRESAHA.121.319994

[18] Rempe RG, Hartz AMS, Bauer B (2016) Matrix metalloproteinases in the brain and blood-brain barrier: versatile breakers and makers. J Cereb Blood Flow Metab 36(9):1481–1507 27323783 10.1177/0271678X16655551 PMC5012524

[19] Zheng X, Ren B, Gao Y (2023) Tight junction proteins related to blood-brain barrier and their regulatory signaling pathways in ischemic stroke. Biomed Pharmacother 165:115272 37544283 10.1016/j.biopha.2023.115272

[20] Cai J, Culley MK, Zhao Y, Zhao J (2018) The role of ubiquitination and deubiquitination in the regulation of cell junctions. Protein Cell 9(9):754–769 29080116 10.1007/s13238-017-0486-3 PMC6107491

[21] Majolee J, Kovacevic I, Hordijk PL (2019) Ubiquitin-based modifications in endothelial cell-cell contact and inflammation. J Cell Sci 132(17) 10.1242/jcs.227728 31488505

[22] Stamatovic SM, Keep RF, Wang MM, Jankovic I, Andjelkovic AV (2009) Caveolae-mediated internalization of occludin and claudin-5 during CCL2-induced tight junction remodeling in brain endothelial cells. J Biol Chem 284(28):19053–19066 19423710 10.1074/jbc.M109.000521 PMC2707189

[23] Drab M, Verkade P, Elger M, Kasper M, Lohn M, Lauterbach B, Menne J, Lindschau C, Mende F, Luft FC, Schedl A, Haller H, Kurzchalia TV (2001) Loss of caveolae, vascular dysfunction, and pulmonary defects in caveolin-1 gene-disrupted mice. Science 293(5539):2449–2452 11498544 10.1126/science.1062688

[24] Razani B, Engelman JA, Wang XB, Schubert W, Zhang XL, Marks CB, Macaluso F, Russell RG, Li M, Pestell RG, Di Vizio D, Hou H Jr., Kneitz B, Lagaud G, Christ GJ, Edelmann W, Lisanti MP (2001) Caveolin-1 null mice are viable but show evidence of hyperproliferative and vascular abnormalities. J Biol Chem 276(41):38121–38138 11457855 10.1074/jbc.M105408200

[25] Jasmin JF, Malhotra S, Singh Dhallu M, Mercier I, Rosenbaum DM, Lisanti MP (2007) Caveolin-1 deficiency increases cerebral ischemic injury. Circ Res 100(5):721–729 17293479 10.1161/01.RES.0000260180.42709.29

[26] Marchiando AM, Shen L, Graham WV, Weber CR, Schwarz BT, Austin JR 2nd, Raleigh DR, Guan Y, Watson AJ, Montrose MH, Turner JR (2010) Caveolin-1-dependent occludin endocytosis is required for TNF-induced tight junction regulation in vivo. J Cell Biol 189(1):111–126 10.1083/jcb.200902153 PMC2854371 20351069

[27] Li X, Garrity AG, Xu H (2013) Regulation of membrane trafficking by signalling on endosomal and lysosomal membranes. J Physiol 591(18):4389–4401 23878375 10.1113/jphysiol.2013.258301 PMC3784187

[28] Zhang M, Chen L, Wang S, Wang T (2009) Rab7: roles in membrane trafficking and disease. Biosci Rep 29(3):193–209 19392663 10.1042/BSR20090032

[29] Gutierrez MG, Munafo DB, Beron W, Colombo MI (2004) Rab7 is required for the normal progression of the autophagic pathway in mammalian cells. J Cell Sci 117:2687–2697 15138286 10.1242/jcs.01114

[30] Edinger AL, Cinalli RM, Thompson CB (2003) Rab7 prevents growth factor-independent survival by inhibiting cell-autonomous nutrient transporter expression. Dev Cell 5(4):571–582 14536059 10.1016/s1534-5807(03)00291-0

[31] Romero Rosales K, Peralta ER, Guenther GG, Wong SY, Edinger AL (2009) Rab7 activation by growth factor withdrawal contributes to the induction of apoptosis. Mol Biol Cell 20(12):2831–2840 19386765 10.1091/mbc.E08-09-0911 PMC2695791

[32] Ceresa BP, Bahr SJ (2006) rab7 activity affects epidermal growth factor:epidermal growth factor receptor degradation by regulating endocytic trafficking from the late endosome. J Biol Chem 281(2):1099–1106 16282324 10.1074/jbc.M504175200

[33] Peralta ER, Martin BC, Edinger AL (2010) Differential effects of TBC1D15 and mammalian Vps39 on Rab7 activation state, lysosomal morphology, and growth factor dependence. J Biol Chem 285(22):16814–16821 20363736 10.1074/jbc.M110.111633 PMC2878074

[34] Roy SG, Stevens MW, So L, Edinger AL (2013) Reciprocal effects of rab7 deletion in activated and neglected T cells. Autophagy 9(7):1009–1023 23615463 10.4161/auto.24468 PMC3722312

[35] Nordmann M, Cabrera M, Perz A, Bröcker C, Ostrowicz C, Engelbrecht-Vandré S, Ungermann C (2010) The Mon1-Ccz1 complex is the GEF of the late endosomal Rab7 homolog Ypt7. Curr Biol 20(18):1654–1659 20797862 10.1016/j.cub.2010.08.002

[36] Kinchen JM, Ravichandran KS (2010) Identification of two evolutionarily conserved genes regulating processing of engulfed apoptotic cells. Nature 464(7289):778–782 20305638 10.1038/nature08853 PMC2901565

[37] Poteryaev D, Datta S, Ackema K, Zerial M, Spang A (2010) Identification of the switch in Early-to-Late endosome transition. Cell 141(3):497–508 20434987 10.1016/j.cell.2010.03.011

[38] Langemeyer L, Borchers AC, Herrmann E, Fullbrunn N, Han Y, Perz A, Auffarth K, Kummel D, Ungermann C (2020) A conserved and regulated mechanism drives endosomal Rab transition. Elife 9 10.7554/eLife.56090 PMC7239660 32391792

[39] Kiontke S, Langemeyer L, Kuhlee A, Schuback S, Raunser S, Ungermann C, Kümmel D (2017) Architecture and mechanism of the late endosomal Rab7-like Ypt7 guanine nucleotide exchange factor complex Mon1–Ccz1. Nat Commun 8(1):14034 28051187 10.1038/ncomms14034 PMC5216073

[40] Jopling HM, Odell AF, Hooper NM, Zachary IC, Walker JH, Ponnambalam S (2009) Rab GTPase regulation of VEGFR2 trafficking and signaling in endothelial cells. Arterioscler Thromb Vasc Biol 29(7):1119–1124 19372461 10.1161/ATVBAHA.109.186239 PMC2704050

[41] Kofler N, Corti F, Rivera-Molina F, Deng Y, Toomre D, Simons M (2018) The Rab-effector protein RABEP2 regulates endosomal trafficking to mediate vascular endothelial growth factor receptor-2 (VEGFR2)-dependent signaling. J Biol Chem 293(13):4805–4817 29425100 10.1074/jbc.M117.812172 PMC5880142

[42] Okon IS, Coughlan KA, Zhang C, Moriasi C, Ding Y, Song P, Zhang W, Li G, Zou MH (2014) Protein kinase LKB1 promotes RAB7-mediated neuropilin-1 degradation to inhibit angiogenesis. J Clin Investig 124(10):4590 – 602. 10.1172/JCI75371 PMC4191012 25180605

[43] Chichger H, Braza J, Duong H, Boni G, Harrington EO (2016) Select Rab GTPases regulate the pulmonary endothelium via endosomal trafficking of vascular Endothelial-Cadherin. Am J Respir Cell Mol Biol 54(6):769–781 26551054 10.1165/rcmb.2015-0286OC PMC4942219

[44] Wang Y, Nakayama M, Pitulescu ME, Schmidt TS, Bochenek ML, Sakakibara A, Adams S, Davy A, Deutsch U, Luthi U, Barberis A, Benjamin LE, Makinen T, Nobes CD, Adams RH (2010) Ephrin-B2 controls VEGF-induced angiogenesis and lymphangiogenesis. Nature 465(7297):483–486 20445537 10.1038/nature09002

[45] Wang Y, Rattner A, Zhou Y, Williams J, Smallwood PM, Nathans J (2012) Norrin/Frizzled4 signaling in retinal vascular development and blood brain barrier plasticity. Cell 151(6):1332–1344 23217714 10.1016/j.cell.2012.10.042 PMC3535266

[46] Arac A, Brownell SE, Rothbard JB, Chen C, Ko RM, Pereira MP, Albers GW, Steinman L, Steinberg GK (2011) Systemic augmentation of alphaB-crystallin provides therapeutic benefit twelve hours post-stroke onset via immune modulation. Proc Natl Acad Sci U S A 108(32):13287–13292 21828004 10.1073/pnas.1107368108 PMC3156222

[47] Jiang SX, Lertvorachon J, Hou ST, Konishi Y, Webster J, Mealing G, Brunette E, Tauskela J, Preston E (2005) Chlortetracycline and Demeclocycline inhibit calpains and protect mouse neurons against glutamate toxicity and cerebral ischemia. J Biol Chem 280(40):33811–33818 16091365 10.1074/jbc.M503113200

[48] Devraj K, Guerit S, Macas J, Reiss Y (2018) An in vivo Blood-brain barrier permeability assay in mice using fluorescently labeled tracers. J Vis Exp (132) 10.3791/57038 PMC5931381 29553506

[49] Peralta ER, Martin BC, Edinger AL (2010) Differential effects of TBC1D15 and mammalian Vps39 on Rab7 activation state, lysosomal morphology, and growth factor dependence**. J Biol Chem 285(22):16814–16821 20363736 10.1074/jbc.M110.111633 PMC2878074

[50] Cantalupo G, Alifano P, Roberti V, Bruni CB, Bucci C (2001) Rab-interacting lysosomal protein (RILP): the Rab7 effector required for transport to lysosomes. EMBO J 20(4):683–693 11179213 10.1093/emboj/20.4.683 PMC145419

[51] Clarkson BD, Ling C, Shi Y, Harris MG, Rayasam A, Sun D, Salamat MS, Kuchroo V, Lambris JD, Sandor M, Fabry Z (2014) T cell-derived Interleukin (IL)-21 promotes brain injury following stroke in mice. J Exp Med 211(4):595–604 24616379 10.1084/jem.20131377 PMC3978271

[52] Li L, Lou W, Li H, Zhu Y, Huang X, Upregulated C-C (2020) Motif chemokine ligand 2 promotes ischemic stroke via chemokine signaling pathway. Ann Vasc Surg 68:476–486 32422289 10.1016/j.avsg.2020.04.047

[53] Lin Y, Zhang JC, Yao CY, Wu Y, Abdelgawad AF, Yao SL, Yuan SY (2016) Critical role of astrocytic interleukin-17 A in post-stroke survival and neuronal differentiation of neural precursor cells in adult mice. Cell Death Dis 7(6):e2273–e2273 27336717 10.1038/cddis.2015.284 PMC5143370

[54] Liu J, Jin X, Liu KJ, Liu W (2012) Matrix metalloproteinase-2-mediated occludin degradation and caveolin-1-mediated claudin-5 redistribution contribute to blood-brain barrier damage in early ischemic stroke stage. J Neurosci 32(9):3044–3057 22378877 10.1523/JNEUROSCI.6409-11.2012 PMC3339570

[55] Underly RG, Levy M, Hartmann DA, Grant RI, Watson AN, Shih AY (2017) Pericytes as inducers of rapid, matrix Metalloproteinase-9-Dependent capillary damage during ischemia. J Neurosci 37(1):129–140 28053036 10.1523/JNEUROSCI.2891-16.2016 PMC5214626

[56] Seaman MNJ, Mukadam AS, Breusegem SY (2018) Inhibition of TBC1D5 activates Rab7a and can enhance the function of the retromer cargo-selective complex. J Cell Sci 131(12) 10.1242/jcs.217398 PMC6031384 29777037

[57] Yu W, Sun S, Xu H, Li C, Ren J, Zhang Y (2020) TBC1D15/RAB7-regulated mitochondria-lysosome interaction confers cardioprotection against acute myocardial infarction-induced cardiac injury. Theranostics 10(24):11244–11263 33042281 10.7150/thno.46883 PMC7532681

[58] Modica G, Lefrancois S (2020) Post-translational modifications: how to modulate Rab7 functions. Small GTPases 11(3):167–173 29099291 10.1080/21541248.2017.1387686 PMC7549697

[59] Kovacs Z, Ikezaki K, Samoto K, Inamura T, Fukui M (1996) VEGF and flt. Expression time kinetics in rat brain infarct. Stroke 27(10):1865–1872 8841346 10.1161/01.str.27.10.1865

[60] Hayashi T, Noshita N, Sugawara T, Chan PH (2003) Temporal profile of angiogenesis and expression of related genes in the brain after ischemia. J Cereb Blood Flow Metab 23(2):166–180 12571448 10.1097/01.WCB.0000041283.53351.CB

[61] Marti HJ, Bernaudin M, Bellail A, Schoch H, Euler M, Petit E, Risau W (2000) Hypoxia-induced vascular endothelial growth factor expression precedes neovascularization after cerebral ischemia. Am J Pathol 156(3):965–976 10702412 10.1016/S0002-9440(10)64964-4 PMC1876841

[62] Ohab JJ, Fleming S, Blesch A, Carmichael ST (2006) A neurovascular niche for neurogenesis after stroke. J Neurosci 26(50):13007–13016 17167090 10.1523/JNEUROSCI.4323-06.2006 PMC6674957

[63] Zhang ZG, Zhang L, Jiang Q, Zhang R, Davies K, Powers C, Bruggen N, Chopp M (2000) VEGF enhances angiogenesis and promotes blood-brain barrier leakage in the ischemic brain. J Clin Invest 106(7):829–838 11018070 10.1172/JCI9369 PMC517814

[64] van Bruggen N, Thibodeaux H, Palmer JT, Lee WP, Fu L, Cairns B, Tumas D, Gerlai R, Williams SP, van Lookeren M, Campagne N, Ferrara (1999) VEGF antagonism reduces edema formation and tissue damage after ischemia/reperfusion injury in the mouse brain. J Clin Invest 104(11):1613–1620 10587525 10.1172/JCI8218 PMC409867

[65] Wilkens M, Holtermann L, Stahl AK, Stegmeyer RI, Nottebaum AF, Vestweber D (2024) Ubiquitination of VE-cadherin regulates inflammation-induced vascular permeability in vivo. EMBO Rep 25(9):4013–4032 39112792 10.1038/s44319-024-00221-7 PMC11387630

[66] Mandel I, Paperna T, Volkowich A, Merhav M, Glass-Marmor L, Miller A (2012) The ubiquitin-proteasome pathway regulates Claudin 5 degradation. J Cell Biochem 113(7):2415–2423 22389112 10.1002/jcb.24118

[67] Li Y, Li S, Wu H (2022) Ubiquitination-Proteasome system (UPS) and autophagy two main protein degradation machineries in response to cell stress. Cells 11(5) 10.3390/cells11050851 PMC8909305 35269473

