# Supplementary material for: Rab7a is required to degrade select blood-brain barrier junctional proteins after ischemic stroke
Source: Acta Neuropathol Commun. 2025 Sep 29;13:203. doi: 10.1186/s40478-025-02125-6 (PMC12482340; doi:10.1186/s40478-025-02125-6)
Supplement: Supplementary file 1 — Supplementary Material 1 [file 40478_2025_2125_MOESM1_ESM.pdf]

Rab7a is required to degrade select blood-brain barrier junctional proteins after ischemic stroke

Azzurra Cottarelli[1,2,*], Danny Jamoul[1*], Mary Claire Tuohy[1], Sanjid Shahriar[3,4,**], Michael Glendinning[1], Grace Prochilo[1], Aimee L. Edinger[5], Ahmet Arac[6,**] and Dritan Agalliu[1,3,7**]

Departments of [1]Neurology, [2]Medicine and [3]Pathology and Cell Biology, Columbia University Irving Medical Center, New York, NY, 10032, USA

[4]Wyss Institute for Biologically Inspired Engineering, Boston, MA 02115, USA

[5]Departments of Developmental and Cell Biology and Pharmaceutical Sciences, University of California, Irvine, CA 92697, USA

[6]Department of Neurology, David Geffen School of Medicine, University of California in Los Angeles, Los Angeles, CA, 90095, USA

[*] These first authors contributed equally to this work.

** These senior authors jointly supervised the work.

7. Corresponding author:

Dritan Agalliu, Ph.D. Email: da191@cumc.columbia.edu

Lead contact:

Dritan Agalliu, Ph.D. Email: da191@cumc.columbia.edu

SUPPLEMENTARY INFORMATION

I. SUPPLEMENTARY FIGURES AND FIGURE LEGENDS

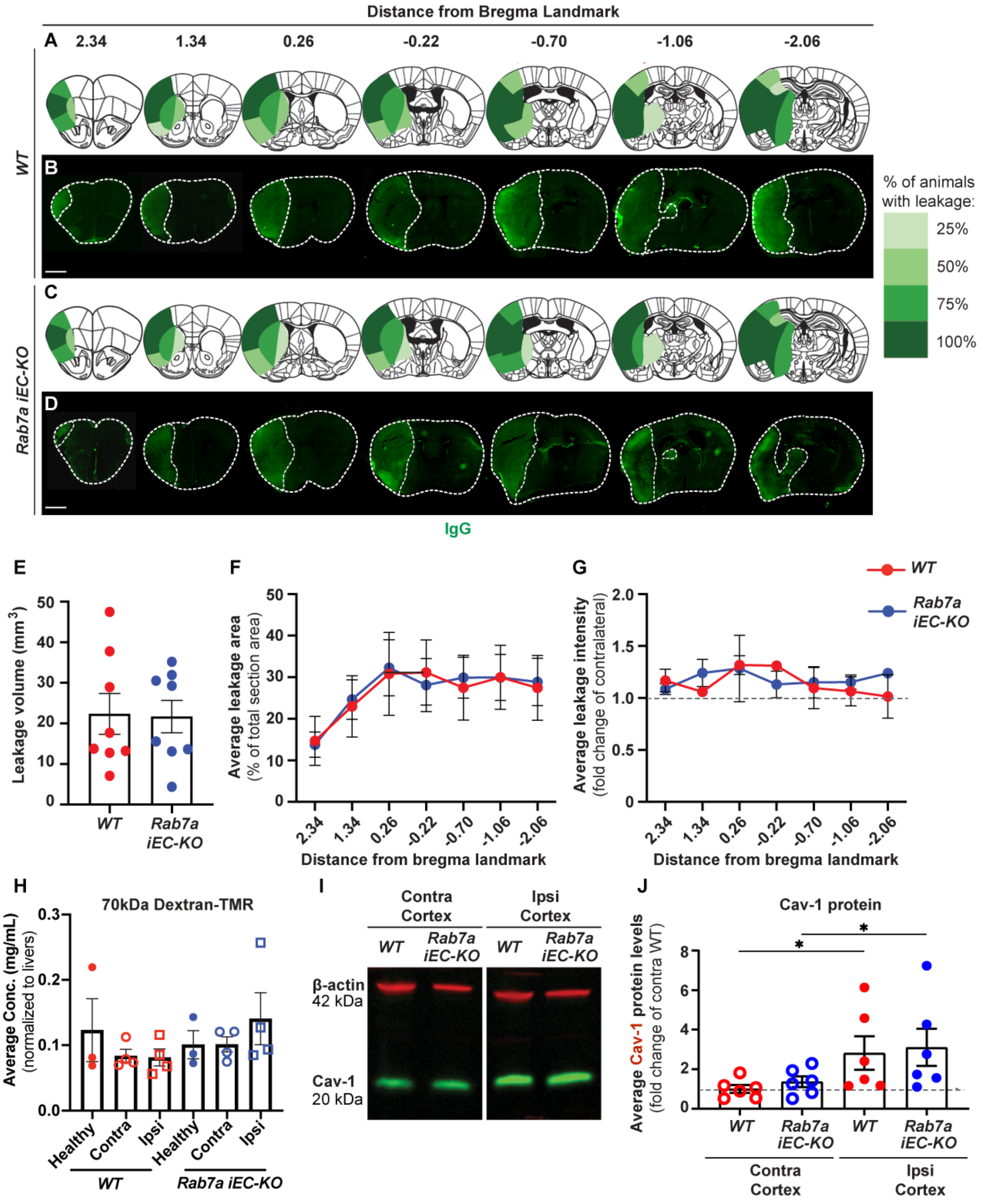

Figure S1. Rab7a elimination in brain endothelial cells does not affect the increase in transcellular BBB permeability 48 hours after t-MCAO.

(**A-D**) Fluorescent micrographs and heatmaps of serum IgG leakage in seven distinct sections at various distances from the bregma landmark of WT (*Rab7a*$^{fl/fl}$) and *Rab7a*^{iECKO} brains 48 hours after t-MCAO. The brain sections were stained for mouse Immunoglobulin G (IgG; green) to detect its leakage into the brain parenchyma (dotted lines outline the brain section borders and serum IgG leakage area). Heatmaps show the fraction of animals displaying serum IgG leakage in each brain section represented as a scale (0-100%) of green hues. (**E**) Quantification of serum IgG leakage volume in WT and *Rab7a*^{iECKO} brains 48 hours after t-MCAO. Each dot represents an animal (n=8; data are means ± s.e.m.; n.s. $p>0.05$ (not shown); Student's t-test). (**F, G**) Quantification of serum IgG leakage area (**F**) and intensity (**G**) in each section at various distances from the bregma landmark in WT and *Rab7a*^{iECKO} brains 48 hours after t-MCAO (Each dot represents the average of n=8 mice / group; data are means ± s.e.m. one-way ANOVA with post-hoc Tukey's correction). (**H**) Quantification of 70 kDa Dextran-TMR leakage in WT and *Rab7a*^{iECKO} ipsilateral and contralateral cortices 48 hours after t-MCAO. Each dot represents an animal [n=3 healthy mice, n=4 mice / genotype for stroke; data are means ± s.e.m.; n.s. $p>0.05$ (not shown); Mann-Whitney t-test]. (**I**) Western blot for Caveolin-1 protein levels in tissue lysates collected from the contralateral and ipsilateral cortices of *WT* and *Rab7a*^{iECKO} brains 48 hours after t-MCAO. (**J**) Quantification of Caveolin-1 protein levels in contralateral and ipsilateral cortical lysates from *WT* and *Rab7a*^{iECKO} brains 48 hours after t-MCAO, normalized to contralateral WT levels. Each dot represents an animal (n=6 mice / group; data are means ± s.e.m.; *: $p<0.05$; n.s.: $p>0.05$ (not shown); one-way ANOVA with post-hoc Tukey's correction).

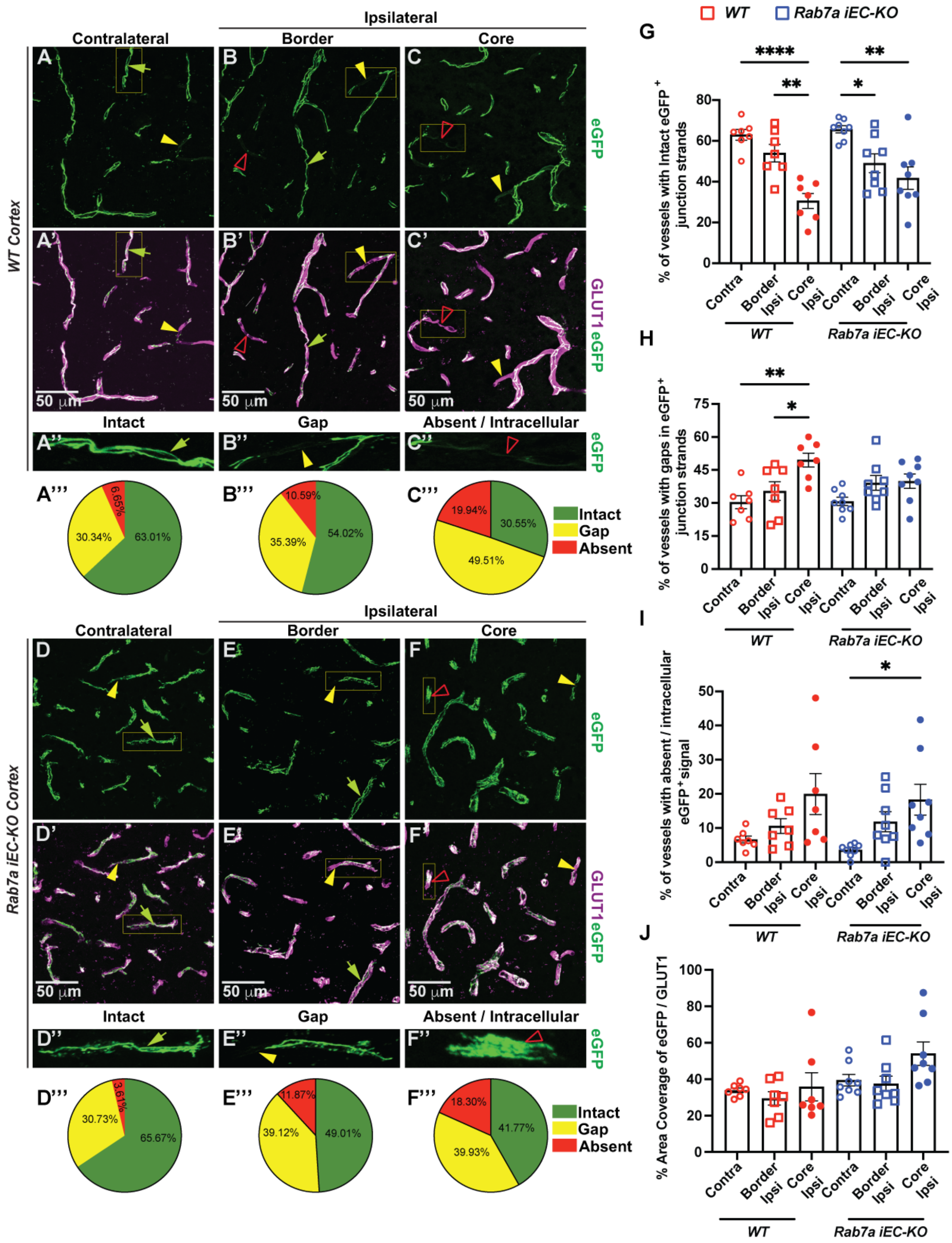
Ipsilateral
Contralateral
Border
Core
WT Cortex
eGFP
GLUT1 eGFP
50 µm
Intact
Gap
Absent / Intracellular
63.01%
30.34%
6.65%
54.02%
35.39%
10.59%
30.55%
49.51%
19.94%
Intact
Gap
Absent
Rab7a iEC-KO Cortex
65.67%
30.73%
3.61%
49.01%
39.12%
11.87%
41.77%
39.93%
18.30%
WT
Rab7a iEC-KO
% of vessels with intact eGFP+ junction strands
% of vessels with gaps in eGFP+ junction strands
% of vessels with absent / intracellular eGFP+ signal
% Area Coverage of eGFP / GLUT1
Contra
Border Ipsi
Core Ipsi

Figure S2. Endothelial Rab7a elimination does not rescue structural abnormalities in eGFP::Claudin-5-positive tight junctions 48 hours after t-MCAO.

(**A-F''')** Immunofluorescence images for eGFP::Claudin5 (green) and Glut1 (magenta) either in the contralateral cortex **(A-A', D-D')**, or border **(B-B', E-E')** and core **(C-C', F-F')** regions of the ipsilateral cortex of *WT* and $Rab7a^{iECKO}$ mice 48 hours after t-MCAO. Normal green arrowhead indicates intact eGFP::Claudin-5^{+} TJ strands, yellow arrowhead points towards gaps in eGFP::Claudin-5^{+} TJ strands, red open arrowheads indicate either absent TJ strands or intracellular eGFP::Claudin-5^{+} aggregates. Scale bar = 50 µm. **(A"-F")** Magnified images of the white boxed areas in **(A-F')** to illustrate the phenotype of the TJ strand. **(A"'- F"')** Pie charts show the percentage of intact (green), gap (yellow), and absent (red) eGFP::Claudin-5^{+} junctional strands present in the contralateral and ipsilateral cortex of *WT* and $Rab7a^{iECKO}$ mice 48 hours after t-MCAO. **(G-I)** Dotted bar graphs of the percentage of vessel segments with (**G**) intact TJ segments, **(H)** TJ strands with gaps, **(I)** absent or intracellular eGFP::Claudin-5^{+} aggregates either in the contralateral cortex, or border and core regions of the ipsilateral cortex of *WT* and $Rab7a^{iECKO}$ mice 48 hours after t-MCAO. **(J)** Dotted bar graph of the percentage of vascular area covered by eGFP^{+} TJ strands. Each dot represents an animal (n= 7/8 mice/group). Data are means ± s.e.m. ***: $p<0.001$; **: $p<0.01$; *: $p<0.05$; one-way ANOVA with post-hoc Tukey's correction.

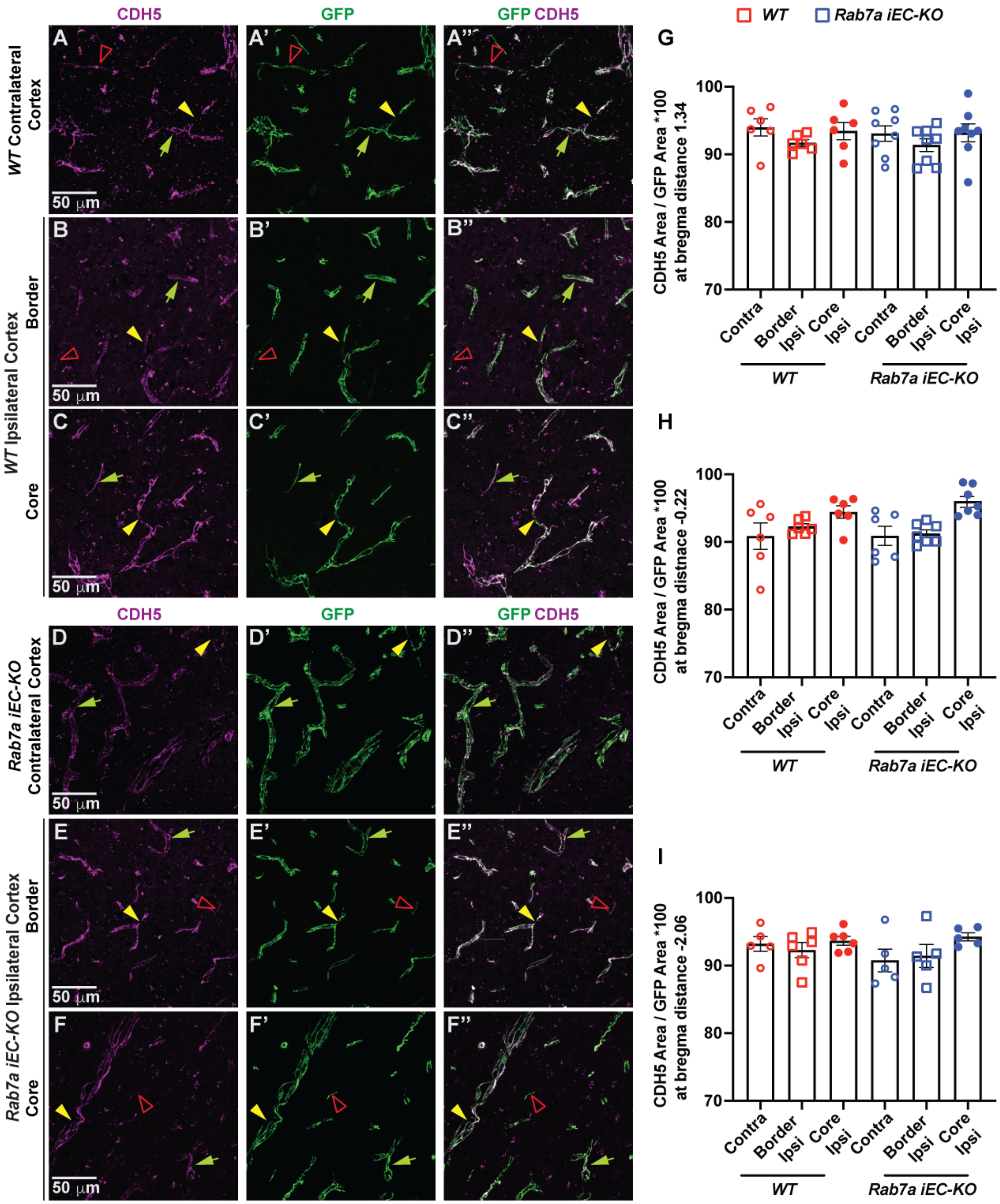
CDH5
GFP
GFP CDH5
WT Contralateral Cortex
WT Ipsilateral Cortex
Border
Core
A
A'
A''
B
B'
B''
C
C'
C''
50 µm
D
D'
D''
E
E'
E''
F
F'
F''
Rab7a iEC-KO Contralateral Cortex
Rab7a iEC-KO Ipsilateral Cortex
WT
Rab7a iEC-KO
G
CDH5 Area / GFP Area *100 at bregma distance 1.34
H
CDH5 Area / GFP Area *100 at bregma distnace -0.22
I
CDH5 Area / GFP Area *100 at bregma distance -2.06
100
90
80
70
Contra
Border Ipsi
Core Ipsi

Figure S3. VE-Cadherin exhibits a similar behavior to eGFP::Claudin-5 in the ipsilateral cortical vessels of *Rab7a^{iECKO}* mice at 48 hours after ischemic stroke.

(A-F'') Immunofluorescences images for VE-Cadherin (CDH5, magenta), and GFP (green) either in the contralateral cortex **(A-A'', D-D'')**, or border **(B-B'', E-E'')** and core **(C-C'',F-F'')** regions of the ipsilateral cortex of *WT* and *Rab7a^{iECKO}* mice expressing the eGFP::Claudin-5 transgene at 48 hours post-t-MCAO. Green arrowheads indicate intact eGFP::Claudin-5^{+} and CDH5^{+} junctional strands, yellow arrowheads point to gaps in eGFP^{+} and CDH5^{+} strands, red open arrowheads indicate either absent, or intracellular eGFP::Claudin-5^{+} and CDH5^{+} junctional strands. Scale bar: 50 µm. (**G-I**) Quantification of the percentage of the area of CDH5 / area of GFP at in three brain sections at distances 1,34 (**G**), -0.22 (**H**) and -2.06 (**I**) from the bregma landmark in the contralateral cortex, or border and core regions of the ipsilateral cortex of *WT* and *Rab7a^{iECKO}* mice at 48 hours after t-MCAO. Each dot represents an animal (n= 6-8 mice/group). Data are means ± s.e.m. There is no significant difference by one-way ANOVA with post-hoc Tukey's correction.

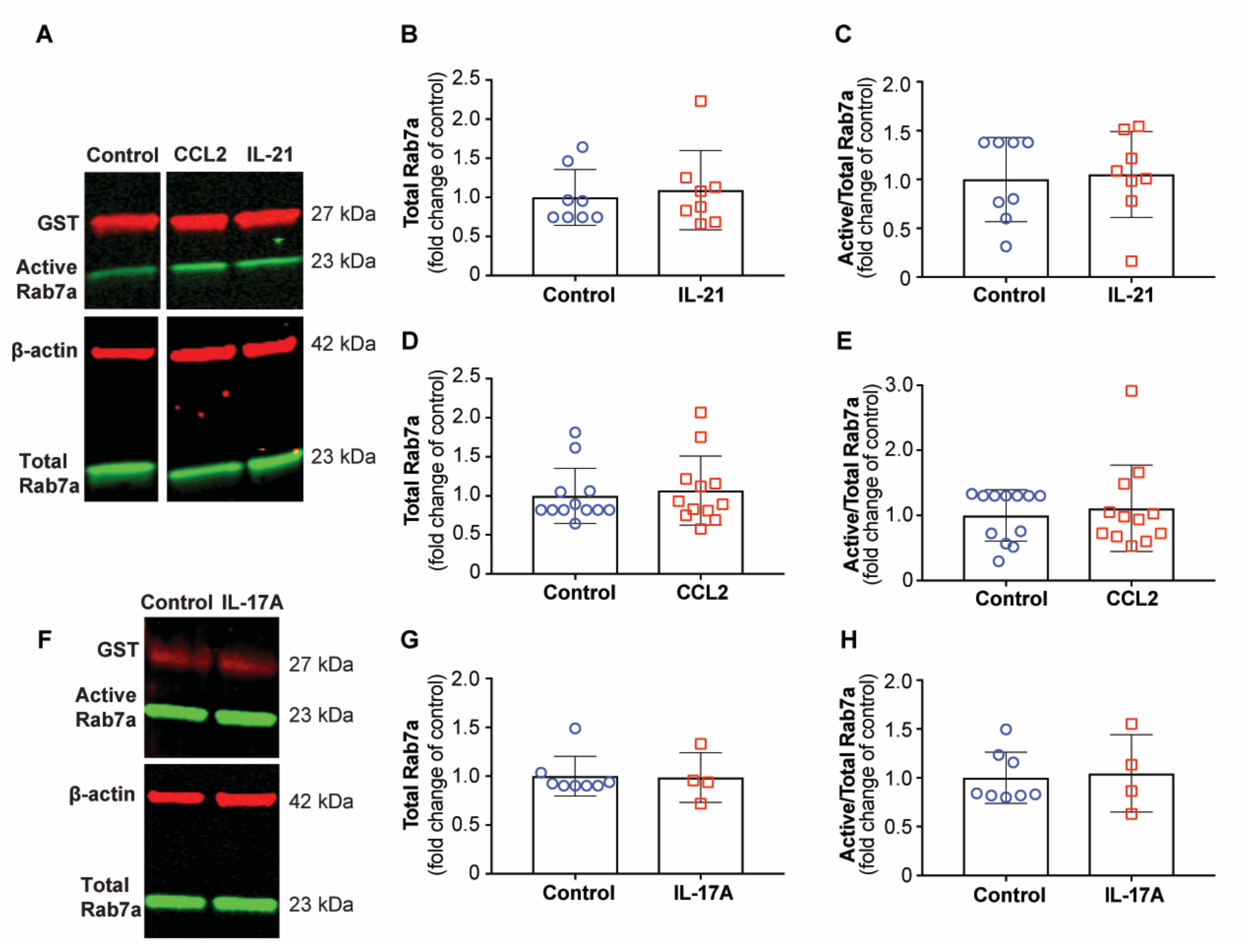

Figure S4. IL-21, CCL2 and IL-17A do not activate Rab7a in primary mouse brain endothelial cells.

(**A, D, G**) Western blot of active and total Rab7a protein levels in primary mouse brain endothelial cells (mBECs) treated with IL-21 (10ng/mL) (**A**), CCL2 (10ng/mL) (**D**) and IL-17A (10ng/mL) (**G**) for 48 hours and untreated control cells. GST and β-actin are used to normalize active and total Rab7a levels, respectively. The molecular weight of each protein is shown on the right. (**B, E, H**) Quantification of total Rab7a protein levels in mBECs treated with IL-21 (**B**), CCL2 (**E**), and IL-17A (**H**) for 48 hours and untreated cells. Each dot represents an independent experiment. (**C, F, I**) Quantification of the ratio of active over total Rab7a protein levels in mBECs treated with

IL-21 (**C**), CCL2 (**F**) and IL-17A (**I**) for 48 hours versus untreated mBECs. Each dot represents an independent experiment (n.s.: p>0.05; Student's t-test).

II. Supplementary Movies

Movie S1. Visualization of ZO-1 gaps in Glut1+ blood vessels in the core region of WT ipsilateral cortex at 48 hours post-t-MCAO. 3D texture-based volume renderings of 12 µm Z stack confocal images on the ipsilateral cortex (core region) of WT mice at 48 hours post-t-MCAO. Glut1+ cells (magenta) label blood vessels and the ZO-1 signal is in green.

Movie S2. Visualization of ZO-1 gaps in Glut1+ blood vessels in the core region of *Rab7a*^{iECKO} ipsilateral cortex at 48 hours post-t-MCAO. 3D texture-based volume renderings of 12 µm Z stack confocal images on the ipsilateral cortex (core region) of *Rab7a*^{iECKO} mice at 48 hours post-t-MCAO. Glut1+ cells (magenta) label blood vessels and the ZO-1 signal is in green.

Movie S3. Visualization of eGFP::Claudin-5 gaps in Glut1+ blood vessels in the core region of WT ipsilateral cortex at 48 hours post-t-MCAO. 3D texture-based volume renderings of 12 µm Z stack confocal images on the ipsilateral cortex (core region) of WT mice at 48 hours post-t-MCAO. Glut1+ cells (magenta) label blood vessels and the eGFP::Claudin-5 signal is in green.

Movie S4. Visualization of eGFP::Claudin-5 gaps in Glut1+ blood vessels in the core region of *Rab7a*^{iECKO} ipsilateral cortex at 48 hours post-t-MCAO. 3D texture-based volume renderings of 12 µm Z stack confocal images on the ipsilateral cortex (core region) of *Rab7a*^{iECKO} mice at 48

hours post-t-MCAO. Glut1+ cells (magenta) label blood vessels and the eGFP::Claudin-5 signal is in green.
